# Discrimination of poisonous and medicinal plants with similar appearance (*Asarum heterotropoides* vs*. Cynanchum paniculatum*) via a fusion method of E-nose, E-tongue, LC-HR-Q-TOF-MS/MS, and electrochemical fingerprint spectra

**DOI:** 10.3389/fchem.2025.1578126

**Published:** 2025-04-29

**Authors:** Xin-Ru Zhang, Yue-Hua Chen, Jia-Nuo Zhang, Wen-Yu Wang, Rui-Bo Sun, Zi-Xuan Ding, Hui Zhang, Ming Xie, Ting-Guo Kang, Hui-Peng Song

**Affiliations:** ^1^ Key Laboratory for Identification and Quality Evaluation of Traditional Chinese Medicine of Liaoning Province, Liaoning University of Traditional Chinese Medicine, Dalian, China; ^2^ Key Laboratory of Ministry of Education for TCM Viscera-State Theory and Applications, Liaoning University of Traditional Chinese Medicine, Shenyang, China

**Keywords:** medicinal plants, electronic nose, electronic tongue, mass spectrometry, Belousov-Zhabotinsky reaction

## Abstract

**Introduction:**

The similarity in appearance of poisonous and medicinal plants, such as Asarum heterotropoides (AH) and Cynanchum paniculatum (CP), poses safety risks due to frequent confusion. Since AH contains toxic ingredients, the traditional methods of olfactory and gustatory identification cannot be used to distinguish AH from CP.

**Methods:**

To differentiate them systematically, we proposed a novel strategy based on dual electronic sensors (DES) and dual fingerprint spectra (DFS). The DES included two intelligent sensors, namely the E-nose and E-tongue, which differentiated AH and CP based on odor and taste, respectively. DFS comprised chemical fingerprint spectra obtained through LC-HR-Q-TOF-MS/MS and electrochemical fingerprint spectra derived from the Belousov-Zhabotinsky reaction, differentiating AH and CP by their specific and overall compositions, respectively. To our knowledge, this was the first time that the E-nose, E-tongue, LC-HR-Q-TOF-MS/MS, and the Belousov-Zhabotinsky reaction were combined to identify AH and CP.

**Results and discussion:**

With the E-nose, we identified 25 major odor components in AH and 12 odor components in CP in a single run of 140 s. Using the E-tongue, bitterness and astringency were identified as their primary taste differences. Furthermore, 91 compounds in AH and 90 compounds in CP were identified through LC-HR-Q-TOF-MS/MS. Both AH and CP shared nitrogenous compounds, volatile oils, organic acids, and lignans. However, AH uniquely contained coumarins and flavonoids, while CP contained steroidal compounds and saccharides. Notably, AH also possessed distinct toxic components, specifically aristolactam I, aristolochic acid D, and safrole. Based on the Belousov-Zhabotinsky reaction, we obtained the electrochemical fingerprint spectra of AH and CP, thereby facilitating further distinction between these two herbs. Through the combination of electrochemical fingerprint spectra with principal component analysis (PCA) or orthogonal partial least squares-discriminant analysis (OPLS-DA), the accuracy of this method reached 100%. Through the fusion strategy, the odors, tastes, components, and electrochemical properties of AH and CP have been systematically analyzed.

## 1 Introduction

Confusion and misuse frequently occur among medicinal plants with highly similar appearances (Xin et al., 2022). This phenomenon not only impacts the efficacy of medications but also poses potential threats to patients’ health. The underground parts of *Asarum heterotropoides* (AH) and *Cynanchum paniculatum* (CP), as two herbal medicines with remarkable medicinal value and highly similar appearances, serve as typical examples of such issues. AH is widely used to treat symptoms such as colds, rhinitis, and coughs, while CP can effectively alleviate stomachaches and toothaches (Zhang et al., 2021). Given their significant differences in pharmacological functions, misusing one for the other can lead to severe consequences. Notably, AH contains poisonous components such as aristolochic acid-like ingredients which has been classified as a Group I cancer-causing agent by the World Health Organization. Its misuse or overdosage can trigger a series of adverse reactions or even lead to life-threatening conditions. Additionally, due to AH’s significantly higher market price compared to CP, some unethical merchants may intentionally adulterate AH with CP for sale. This further poses challenges to the authentication of these two medicinal plants. To effectively prevent the confusion and misuse, there is an urgent need to adopt modern technologies and establish reliable strategies to discriminate them from multiple angles.

Electronic sensory technologies have demonstrated unique advantages in the identification of medicinal plants. Electronic nose (E-nose) and electronic tongue (E-tongue) are two representative electronic sensory technologies (Tibaduiza et al., 2024; Wang S. et al., 2022). The E-nose perceives and analyzes volatile odors by simulating the human olfactory system. Using E-nose, Zhang et al. differentiated raw *Magnolia officinalis* and ginger-processed *M. officinalis* and identified 16 possible odor components (Zhang et al., 2022). Lu et al. employed E-nose combined with gas chromatography-mass spectrometry to identify 40 aroma components from chamomile (Lu et al., 2024). In another example, the adulterants and geographical origins of *Ziziphi Spinosae* were successfully identified by E-nose and headspace gas chromatography-mass spectrometry (Zhang et al., 2023). Similarly, as an intelligent taste recognition tool, the E-tongue has also been widely applied in the field of medicinal plants. For example, Lei et al. conducted comprehensive evaluations of the aroma and taste of bear bile powder and its common counterfeit by E-nose and E-tongue technologies (Lei et al., 2023). Xing et al. determined the taste characteristics of *Polygonum multiflorum* using E-tongue and revealed the relationship between tastes and components (Xing et al., 2021). Wang et al. studied the correlation between the fragrance, taste, and effective components of *Gastrodiae Rhizoma* by E-nose and E-tongue (Wang B. et al., 2022). In summary, the rapid development of E-nose and E-tongue technologies provides a new approach for the identification of morphologically similar medicinal plants.

Chemical fingerprint spectra based on liquid chromatography-mass spectrometry (LC-MS) is one of the effective strategies for the analysis of chemical components in medicinal plants (Chen et al., 2021; Liang et al., 2022). In recent years, this technique has been increasingly and widely applied in this field. For instance, Bao et al. revealed at least 18 different chemical components in *Coptidis Rhizoma* by using UPLC-Q/TOF-MS (Bao et al., 2024). Mei et al. identified 50 components in *Spatholobi Caulis* by LC-Triple TOF-MS (Mei et al., 2021). Batsukh et al. utilized LC-IT-TOF-MS/MS in conjunction with multivariate statistical analysis to identify 30 compounds from *Divaricate Saposhnikoviae* (Batsukh et al., 2020). With this method, researchers can obtain detailed fingerprint spectra and abundant information on chemical components. Although LC-MS is effective in the identification of medicinal plants, it comes with drawbacks like expensive equipment and lengthy data analysis. In recent years, electrochemical fingerprint spectra has emerged and developed rapidly (Lan et al., 2023). Compared to LC-MS, electrochemical fingerprint spectra offers advantages including cheap instrumentation, simple sample treatment and short detection time, making it an effective complement to LC-MS. Furthermore, it can intuitively reflect the overall characteristic information of medicinal plants. The principle indicates that during the electrochemical reaction process, the chemical components in different medicinal plants will elicit unique changes, leading to characteristic fingerprint spectra. Zeng et al. utilized fingerprint spectra on the basis of three-electrode system to differentiate *Coptidis Rhizoma* from its adulterants (Zeng and Jiang, 2022). Tarighat et al. used fingerprint spectra based on cyclic voltammetry to classify and identify Lamiaceae herbs such as mint and lavender (Tarighat et al., 2023). Liu et al. discovered significant differences in the fingerprint spectra of *Astragali Radix* from various provinces through differential pulse voltammetry (Liu and Yan, 2023). However, electrochemical fingerprint spectra commonly identify medicinal plants from the overall components, lacking specificity for individual components. It seems that the combination of LC-MS and electrochemical fingerprint spectra is an ideal method for the analysis of medicinal plants. Currently, there are few reports on the combination of the two methods.

In this study, a novel strategy combining dual electronic sensors (DES) and dual fingerprint spectra (DFS) was proposed for differentiating AH and CP. This strategy emphasized the integration of electronic sensory technology and fingerprint spectra analysis. On the one hand, E-nose and E-tongue were utilized to differentiate AH and CP from the perspectives of odor and taste, respectively. On the other hand, chemical fingerprint spectra obtained through LC-HR-Q-TOF-MS/MS and electrochemical fingerprint spectra derived from the Belousov-Zhabotinsky reaction were employed to differentiate AH and CP, focusing on specific chemical components and overall characteristic information, respectively. Furthermore, the electrochemical fingerprint spectra was combined with PCA and OPLS-DA to ensure a 100% accurate differentiation between AH and CP. Through the implementation of the DES and DFS strategy, a comprehensive and systematic differentiation of AH and CP from multiple angles was achieved.

## 2 Materials and methods

### 2.1 Reagents and materials

Seven different batches of AH were purchased from the regional medicinal herb trading center of Anguo City, Hebei Province (batches: 07230307, 07230401, 07230504, 07230604, 07230702, 07230801, 07230905). Seven different batches of CP were purchased from the regional medicinal herb trading center of Lu’an City, Anhui Province (batches: 23060201, 23070304, 23080302, 23090203, 23100402, 23110501, 23120204). The chemical standards including asarinin (PS010871), methyl eugenol (PS001191), (1R)-(+)-α-pinene (PS230925-10), (+)-3-carene (PS230926-01), eucalyptol (PS020906), carvacrol (PS230925-13), α-terpineol (PS020226), paeonol (PS000281), hesperidin (PS010632), chlorogenic acid (PS010694), o-hydroxyacetophenone (PS230925-14), p-hydroxyacetophenone (PS020038), palmitic acid (PS020930), and oleic acid (PS020507) were all purchased from Chengdu Push Bio-technology Co., Ltd. Vanillic acid (MUST-23012113), caffeic acid (MUST-23061118), and (−)-β-pinene (MUST-2392216) were purchased from Chengdu Must Biotechnology Co., Ltd. The purity of all the above compounds was above 98%. Purified water was purchased from Wahaha Group Co., Ltd. (Hangzhou, China). H_2_SO_4_ (20111014) was purchased from Sinopharm Chemical Reagent Co., Ltd. CH_2_(COOH)_2_ (M813041) was purchased from Macklin Co., Ltd. (NH_4_)_2_SO_4_·Ce(SO_4_)_2_ (20230601) was purchased from Tianjin Damao Chemical Reagent Co., Ltd. KBrO_3_ (20160107) and LC-grade methanol (20241101) were purchased from Tianjin Kermel Chemical Reagent Co., Ltd. LC-grade acetonitrile (JB145430) and MS-grade formic acid (20171008) were purchased from Merck (Darmstadt, Germany).

### 2.2 Sample preparation

The roots and rhizomes of AH and CP were powdered and passed through a sieve. Then 0.5 g of sample was weighed and mixed with 10 mL of methanol for a 40-min ultrasonic extraction (F-050 type, Fuyang ultrasonic cleaner). After centrifugation (LC-LX-H185C type, Lichen Co., Ltd.) at 14,000 rpm for 5 min, the supernatant was used for LC-HR-Q-TOF-MS/MS analysis. Another 0.5 g of sample powder was weighed and mixed with 10 mL of deionized water for a 30-min ultrasonic extraction. The extract was filtered and diluted tenfold for E-tongue analysis. The powders of medicinal plants were directly used for electrochemical analysis. Prior to E-nose analysis, both dried AH and CP samples were processed into uniform small segments (1 cm in length) to ensure morphological standardization. Each headspace vial was filled with 1.0 g of the processed sample material. This standardization procedure aimed to unify both morphology and mass, thereby reducing variations in the detection of volatile components.

### 2.3 Setup and conditions of E-nose

The E-nose (Alpha MOS SA Heracles NEO) was employed for analysis, equipped with an automatic sampling device, an ultra-fast gas chromatography (GC) unit, two flame ionization detectors (FID), and two columns of different polarities (MXT-5 and MXT-1701). The volume of a headspace vial was 20 mL and the sample weight was 1.0 g. Seven batches of samples were prepared and each sample was subjected to three replicate measurements. The injection volume was set at 4,000 μL, with an incubation temperature of 60°C and an incubation time of 20 min. The injection speed was 125 μL/s, lasting for 45 s. The inlet temperature was maintained at 200°C, while the trap temperature was set at 40 °C. Hydrogen was used as the carrier gas at a flow rate of 1.0 mL/min. The trap time was 50 s, and the final temperature of the trap was 240°C. The initial column temperature was 50°C. The temperature was programmed to ramp up from 0.5°C/s to 90°C, followed by an increase of 4°C/s to 250°C, where it was held for 15 s. The acquisition time was 137 s, and the FID gain was set at 12. A mixture of n-alkanes (C_6_–C_16_) was used as the chemical reference.

### 2.4 Setup and conditions of E-tongue

The sensors (AAE, CT0, CA0, C00, AE1) and reference electrodes of the E-tongue (INSET Intelligent Sensor Technology, Inc. Taste Sensing System SA402B) were separately immersed in the reference solution (30 mmol/L potassium chloride and 0.3 mmol/L tartaric acid) and 3.33 mol/L potassium chloride solution for 24 h for activation. Calibration was performed using the reference solution, followed by the measurement of the umami, saltiness, sourness, bitterness, astringency, and richness of seven batches of samples at a room temperature of 25°C. After a brief rinse with the reference solution, sensors C00 and AE1 were used to determine residual tastes (bitter and astringent aftertaste). The data acquisition time was 30 s, with a total of 4 cycles collected. Due to significant fluctuations in the data from the first cycle, this cycle’s data was excluded from the analysis. Data from the second to fourth cycles were retained. Each batch was analyzed in triplicate, and the mean of triplicate measurements was adopted for subsequent analysis.

### 2.5 Conditions of LC-HR-Q-TOF-MS/MS

The chemical compositions of AH and CP were analyzed using an Agilent LC-6500 series Q-TOF liquid chromatography-mass spectrometry system. The separation column was an Agilent Phenyl-Hexyl column (4.6 × 50 mm, 3.5 μm). The mobile phase consisted of 0.1% formic acid in water (A) and acetonitrile (B). The flow rate was set at 0.5 mL/min. The injection volume was 1 μL. Gradient elution was performed as follows: 0–8 min, 5%–5% B; 8–20 min, 5%–20% B; 20–40 min, 20%–60% B; 40–45 min, 60%–95% B; 45–50 min, 95%–95% B. The Q-TOF-MS/MS system was used for analysis in positive or negative ion mode. The operational parameters were set as follows: drying gas temperature at 200°C, drying gas flow rate at 11 L/min, nebulizer gas pressure at 35 psi, sheath gas temperature at 350°C, sheath gas flow rate at 8 L/min, capillary voltage at 4,000 V, *m/z* range from 100 to 1,000, nozzle voltage at 1,000 V, fragmentation voltage at 120 V, and collision energy at 30 eV. Auto MS/MS was used for data acquisition. Compounds with available chemical reference standards could be accurately identified by comparing their retention times, molecular ions, and secondary fragment ions with those of the reference standards. For unknown compounds, a combined approach that utilized both self-built compound libraries and public compound databases was employed for structural elucidation. On one hand, a mass spectrometry database for AH and CP was compiled from relevant literature, encompassing chemical formulas, molecular ions, fragment ions, and other related parameters (Wang et al., 2023; Wen et al., 2014; Zhang et al., 2021; Mao et al., 2017; Gao et al., 2019; Chen et al., 2023; Hu et al., 2025; Yu et al., 2016). On the other hand, public databases such as PubChem, METLIN, ChemSpider, and mzCloud spectral library were used for the structural comparison of the compounds.

### 2.6 Setup and conditions of electrochemical fingerprint spectra

The Belousov-Zhabotinsky reaction was conducted in a continuously stirred reactor (85–2 type, Changzhou Yuexin Instrument Manufacturing Co., Ltd.). A graphite electrode was used as the reference electrode, and a platinum electrode was used as the indicator electrode. To the reactor, 0.4 g of sample powder, 24 mL of H_2_SO_4_ solution (3 mol/L), 12 mL of CH_2_(COOH)_2_ solution (1 mol/L), and 6 mL of (NH_4_)_2_SO_4_·Ce(SO_4_)_2_ solution (0.1 mol/L) were added. The temperature of the reaction system was controlled at 310 K. After stirring at a constant speed of 600 r/min for 5 min, 6 mL of KBrO_3_ solution (0.2 mol/L) was rapidly injected through a syringe to initiate the reaction. Immediately, the data acquisition program was started to record the electrochemical fingerprint spectrum until the oscillation of electric potential disappeared.

### 2.7 Statistical analysis

To analyze Q-TOF data, Agilent MassHunter was employed. The Heracles NEO E-nose was controlled via Alpha Soft, wherein principal component analysis (PCA) and discriminant factor analysis (DFA) were implemented for data processing. The AroChemBase database was used to identify volatile compounds and obtain sensory description. Origin 2021 was utilized to generate fingerprint spectra for E-nose, radar charts for E-tongue, and electrochemical fingerprint spectra for Belousov-Zhabotinsky reaction. GraphPad Prism 6 was applied to generate box plots. SIMCA was utilized to perform PCA and orthogonal partial least squares discriminant analysis (OPLS-DA).

## 3 Result and discussion

### 3.1 Technical route

**FIGURE 1:**
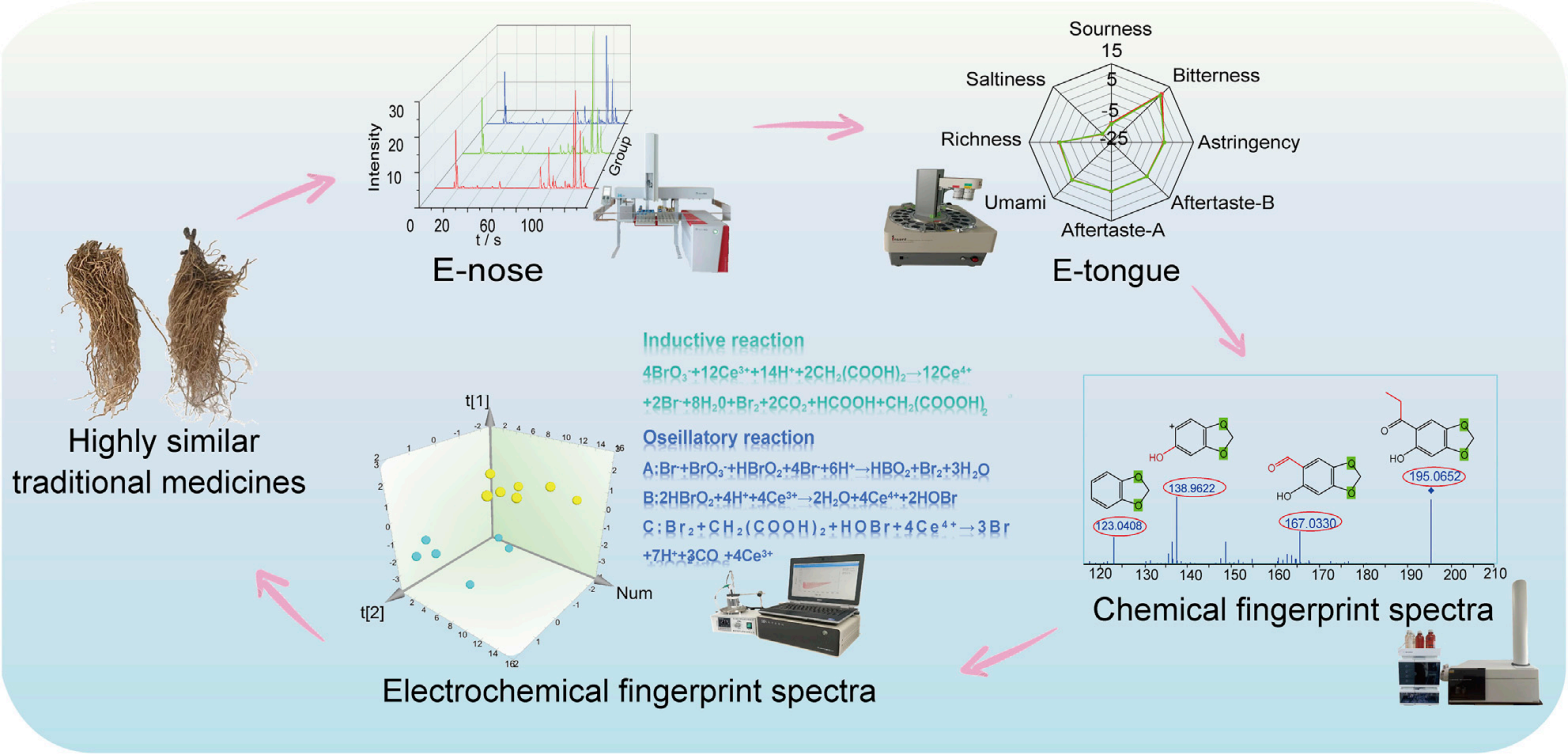
A four-step technical route for differentiation of AH and CP.

AH and CP are commonly used but easily confused medicinal plants due to their highly similar appearance. Due to the presence of toxic ingredients in AH, the conventional methods of identification through smell and taste cannot be employed to differentiate AH from CP. To achieve this, a four-step technical route utilizing dual electronic sensors (DES) and dual fingerprint spectra (DFS) was proposed for the first time (Figure 1). Firstly, an E-nose was applied to capture characteristic gas information, with PCA and DFA adopted to distinguish them further. Secondly, an E-tongue was utilized to obtain characteristic tastes, with radar charts and box plots used to analyze their taste differences. The strategy of DES could overcome the shortcomings of the traditional methods of olfactory and gustatory identification which could not be used for the identification of toxic medicinal plants. Thirdly, LC-HR-Q-TOF-MS/MS was employed to analyze the differences in chemical compositions between AH and CP, yielding their chemical fingerprint spectra. Fourthly, the Belousov-Zhabotinsky reaction was utilized to acquire electrochemical fingerprint spectra, differentiating them from the perspective of electrochemical properties. By integrating E-nose, E-tongue, chemical fingerprint spectra, and electrochemical fingerprint spectra, a systematic and multi-angled differentiation between AH and CP was achieved.

### 3.2 E-nose analysis

**FIGURE 2:**
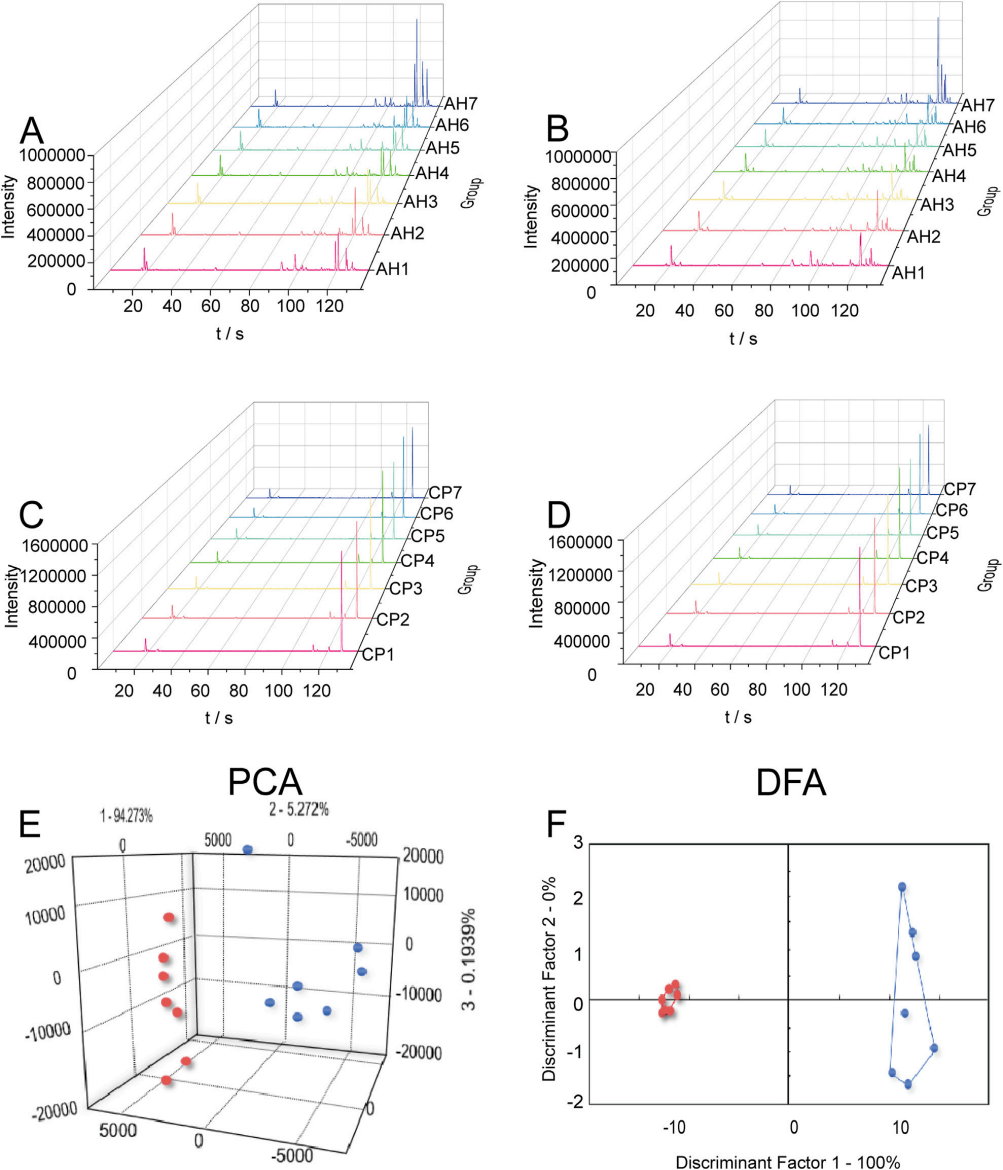
Odor chromatograms of AH on MXT-5 **(A)** and MXT-1701 **(B)**. Odor chromatograms of CP on MXT-5 **(C)** and MXT-1701 **(D)**. PCA **(E)** and DFA **(F)** for distinguishing between AH and CP based on E-nose.

Using the Heracles NEO ultra-fast gas-phase E-nose, odor chromatograms for AH and CP were established on two types of chromatographic columns: MXT-5 and MXT-1701 (Figure 2). It was evident that each sample could be analyzed within 140 s, demonstrating remarkable efficiency. Through comparison with the Arochembase database, 25 odor components were identified in AH and 12 odor components in CP. Table 1 provides detailed information on the compounds and their odor descriptions. Five compounds, including camphene, tridecane, 2,2,4-trimethylpentane, limonene, and acetaldehyde, were found to be shared by AH and CP. These compounds were associated with a range of odor descriptions, such as freshly cut grass, fruity, aromatic, spicy, alkane-like, acidic, and petrol-like notes. The unique components in AH, such as pyridine, butylbutanoate, o-chlorotoluene, pentadecane, and butan-2-one, were primarily characterized by notes of freshly cut grass, alkane-like qualities, and spicy aromas. CP contained unique components like gamma-decalactone, hexane, (Z)-3-hexenal, alpha-ionone, and cymen-8-ol, which exhibited petrol-like, greasy, and sweet odors. While there were similarities in the odor descriptions of AH and CP, they also possessed distinct characteristics that set them apart.

**TABLE 1:** Possible compounds and sensory descriptions of AH and CP.

	Molecular formula	Reserved parameter		Possible compound	Correlation index	Sensory description	AH	CP
		MXT-5	MXT-1701					
1	C_11_H_24_	1098	1097	Terpinolene	98.92	Star anise; oranges; fresh fruits; herbaceous plant; pine tree, plastic, sweet, woody scent	+	
2	C_10_H_18_O_2_	1474	1688	Gamma-decalactone	98.76	Coconut; greasy; fresh; fruity (dry); lactones; greasy; oily (fresh); peach; sweet; candlesmell		+
3	C_15_H_32_	1486	1488	Pentadecane	96.78	Alkane; heteroalcohols; freshly mowed	+	
4	C_12_H_24_O_2_	1417	1488	Methy lundecanoate	96.24	Brandy; greasy; fruits; greasy; sweet; thesmellofcandles; wine	+	
5	C_10_H_22_	953	963	4-ethyl-octane	95.84		+	
6	C_12_H_26_	1146	1130	Decane	95.47	Oak; apple; greasy; fruits; grass; freshly mowed; luxuriant		+
7	C_6_H_10_O	802	893	(Z)-3-hexenal	95.41			+
8	C_10_H_16_O	1147	1288	Camphor	94.61	Greasy; freshly mowed; greenpepper; mushrooms; pepper; butter	+	+
9	C_13_H_20_O	1405	1559	Alpha-ionone	94.43	Fragrant with oil or spices; cedar; floral or botanical; fruits; dovetail; sweet; tropical; the violet; warm; woody scent		+
10	C_5_H_10_O	664	740	2-methyl butanal	94.32	Almonds; apple; charred; burning (strong); asphyxiating; coco; coffee; fermented or brewed; fruits; freshly mowed; iodoform; malt; musty smell; nutty; powerful; agreasy smell of incense; acidity	+	
11	C_13_H_28_	1307	1286	Tridecane	94.27	Alkane; oranges; fruits; heteroalcohols; hydrocarbon	+	+
12	C_4_H_8_O	600	693	Butan-2-one	94.16	Acetone; butter; cheese; chemistry; chocolate; the atmosphere; aromatic; fruits; gaseous; cheerful; spicy; sharp; sweet	+	
13	C_5_H_8_O_2_	943	1097	4-pentanolide	93.90	Fennel; coco; herbaceous; sweet; tobacco; warm; woody scent	+	
14	C_3_H_6_O	459	559	Propanal	93.85	Acetaldehyde; coco; earthy; the atmosphere; nutty; plastics; spicy; solvent	+	
15	C_10_H_14_O	1182	1338	Cymen-8-ol	93.60	Cherry; oranges; coumarin; floral or botanical; fruits; fruity (sweet); musty smell; sweet		+
16	C_8_H_18_	683	681	2,2,4-trimethyl pentane	93.27	Gasoline	+	+
17	C_6_H_14_O	802	893	2-hexanol	93.05	Cauliflower; chemistry; greasy; fruits; terpene; wine	+	
18	C_7_H_10_O_3_	1190	1445	5-ethyl-3-hydroxy-4-methyl-2(5H)-furanone	92.35	Brown sugar; butterscotch; caramel; fruits; fruity (sweet); maple; nutty; condiments; spicy; sweet	+	
19	C_10_H_16_	1037	1078	Limonene	91.55	Oranges; freshly mowed; pine tree	+	+
20	C_5_H_12_	518	485	Pentane	87.72	Alkane; gasoline		+
21	C_8_H_18_	769	789	3-methylheptane	86.20	Green plants; sweet	+	
22	C_10_H_10_O_2_	1407	1560	(E)-methyl cinnamate	83.67		+	
23	C_13_H_26_O_2_	1533	1607	Methyl dodecanoate	83.01	Coconut; creamy; greasy; floral or botanical; fruits; mushrooms; soap; sweet; the smell of candles; waxy	+	
24	C_10_H_12_O_2_	1244	1358	Ethyl phenylacetate	82.62	Fennel; cinnamon; coco; floral or botanical; fruits; honey; rose; spicy; sweet; candlesmell	+	
25	C_2_H_4_O	433	499	Acetaldehyde	81.05	Aldehyde group; the atmosphere; fresh; fruits; cheerful; piquant	+	+
26	C_10_H_16_	995	1069	Alpha-phellandrene	73.02	Orange; freshly cut grass scent; mint flavor; spicy; terpene aroma; pine resin; woody scent	+	
27	C_10_H_16_	995	1069	Myrcene	72.96	Sesame oil aroma; spice fragrance; airy; fruity; geranium; lemon; metallic; musty; plastic; pleasant; resinous; soapy; spicy; sweet; woody scent	+	
28	C_10_H_16_	995	1069	(+)-alpha-phellandrene	72.65	Dill flavor	+	
29	C_15_H_24_	1577	1627	1-phenyl-nonane	77.81		+	
30	C_9_H_20_	928	873	Nonane	76.80	Alkane; heteroalcohols; gasoline		+
31	C_10_H_18_O	1235	1358	3-decen-2-one	68.65	Unctuous	+	
32	C_3_H_6_O_2_	484	596	Methy lacetate	52.09	Blackcurrant; the atmosphere; aromatic; fruits; fruity (sweet); cheerful; solvent; sweet	+	

To further distinguish AH from CP, the chromatographic peaks obtained by the E-nose were used as influencing factors for PCA (Figure 2E) and DFA (Figure 2F). In the PCA model, the first principal component (PC1) contributed 94.183%, while the second principal component (PC2) contributed 4.729%. The cumulative contribution rate of the principal components reached 98.912%, indicating that AH and CP could be well distinguished. In the DFA model, the horizontal and vertical coordinates represented the first discriminant factor (DF1) and the second discriminant factor (DF2), respectively. The DF1 in Figure 2F was 100%, suggesting that DFA could better distinguish AH and CP samples based on odor characteristics. The result demonstrated that the E-nose combined with DFA was effective to distinguish AH from CP from the perspective of odor. The efficiency of this method was demonstrated in two aspects. On the one hand, plant samples used for E-nose analysis did not require grinding and extraction, thus offering significant advantages in sample pretreatment. On the other hand, the single analysis time for each plant sample was 140 s, which significantly shortened the analysis time compared to traditional methods.

### 3.3 E-tongue analysis

**FIGURE 3:**
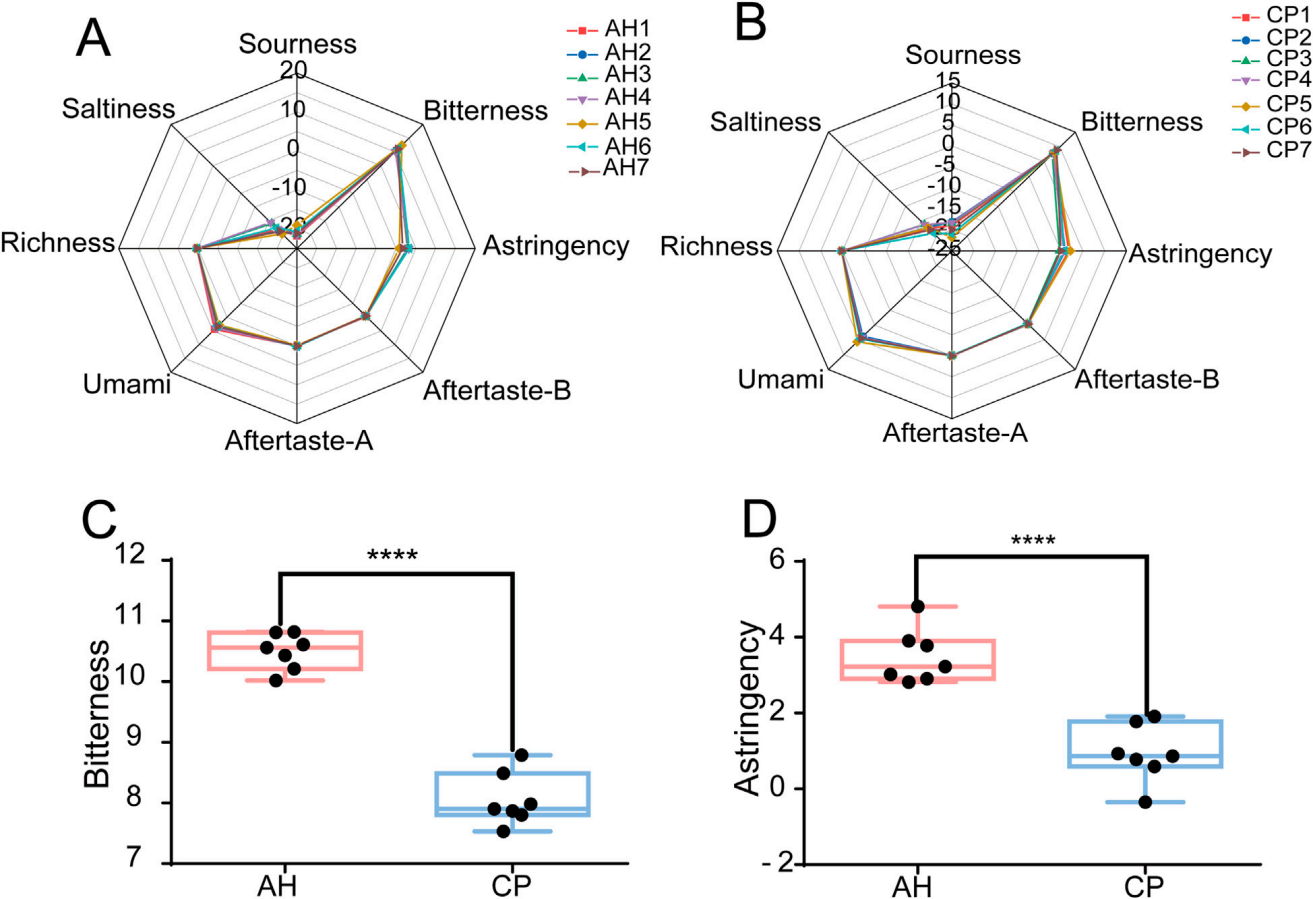
Electronic tastes of AH **(A)** and CP **(B)**. Comparison of bitterness **(C)** and astringency **(D)** between AH and CP.

The taste values of AH and CP samples were measured using an E-tongue, and radar charts were constructed based on the signals collected by the sensors (Figures 3A,B). While an initial observation suggested a similar overall shape in the radar charts, a closer analysis revealed significant differences (P < 0.05) between AH and CP in terms of bitterness, astringency, sourness, aftertaste-A, and richness, as shown in Figures 3C,D and Supplementary Figure S1. The taste response range of the E-tongue encompasses the following: sourness (−13 to 12), bitterness (0–25), astringency (0–25), and saltiness (−6–19). Importantly, only values falling within these ranges could reflect the corresponding taste. Notably, the bitterness and astringency of AH were significantly higher than those of CP, suggesting that these two tastes could serve as key discriminators between the two samples.

**FIGURE 4:**
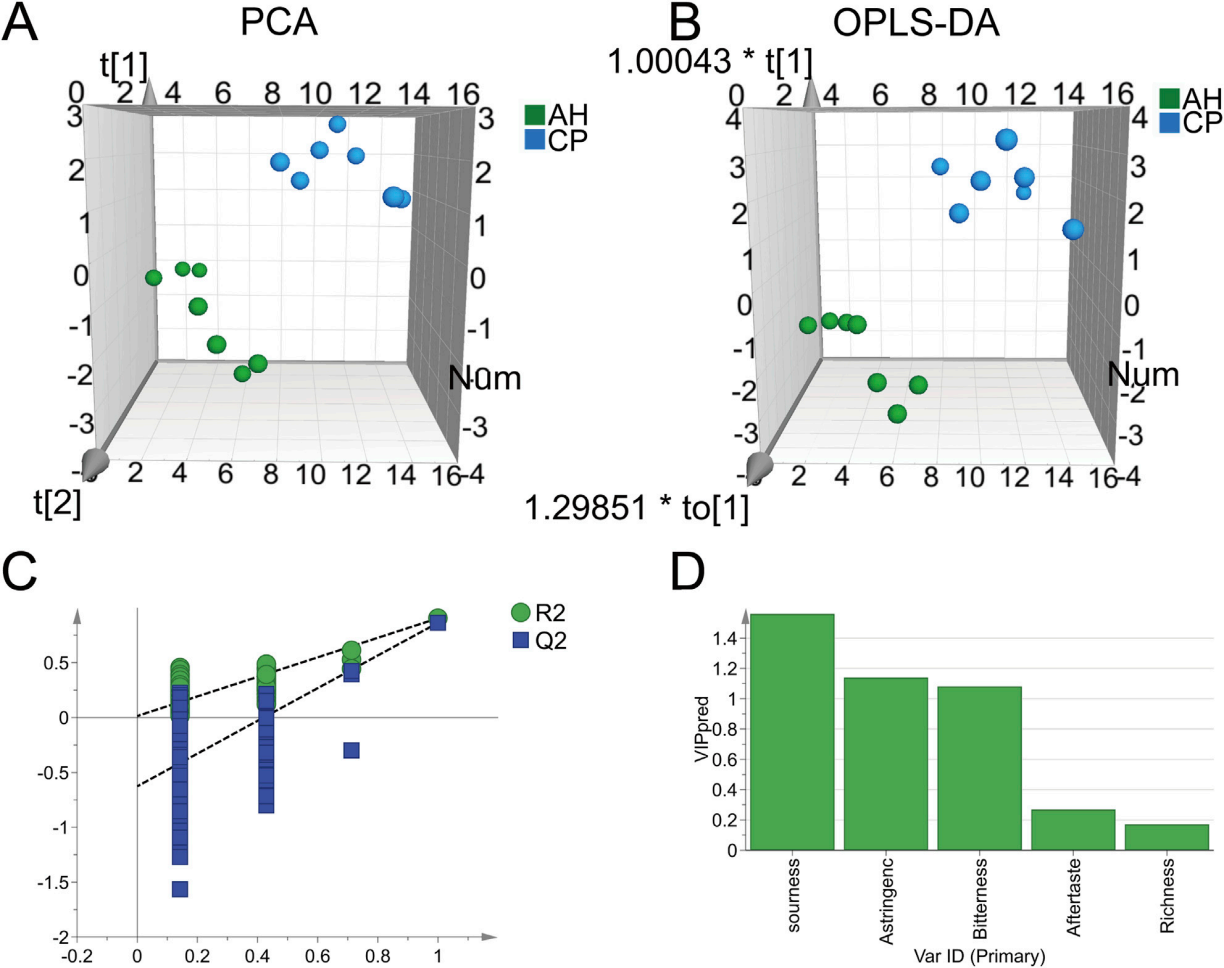
PCA **(A)** and OPLS-DA **(B)** of AH and CP based on electronic tastes. The permutation test **(C)** and VIP values **(D)** of OPLS-DA.

PCA and OPLS-DA were employed to further differentiate between AH and CP. As shown in Figures 4A,B, the samples of AH and CP could be clearly separated from each other. The PCA model achieved R^2^ and Q^2^ values of 0.869 and 0.607, respectively. Furthermore, the OPLS-DA model demonstrated R^2^X, R^2^Y, and Q^2^ values of 0.93, 0.936, and 0.895, respectively. These parameters confirm the reliability of the results. To further validate the credibility of the model, a permutation test was performed (Figure 4C). Ideally, the R^2^Y intercept and Q^2^Y intercept of a valid model should not have exceeded 0.4 and 0.05, respectively (Wang et al., 2024). In this case, the R^2^Y intercept and Q^2^Y intercept were 0.113 and −0.666 respectively, indicating the results were credible. A VIP value greater than 1 was considered a criterion for differential variables (Wang et al., 2024). As shown in Figure 4D, the VIP values for bitterness and astringency exceeded this threshold, which was consistent with the previous findings. Consequently, the results suggested that the E-tongue could effectively differentiate between AH and CP from the perspective of tastes. The necessity of employing E-tongue analysis was underscored by two aspects: (1) AH contained toxic components such as aristolochic acid-like ingredients, thus traditional taste-testing methods could lead to poisoning; (2) The taste of CP was unpleasant and nauseating, making taste-testing methods unsuitable as well.

### 3.4 Chemical fingerprint spectra based on LC-HR-Q-TOF-MS/MS

#### 3.4.1 Identification of chemical compositions

**FIGURE 5:**
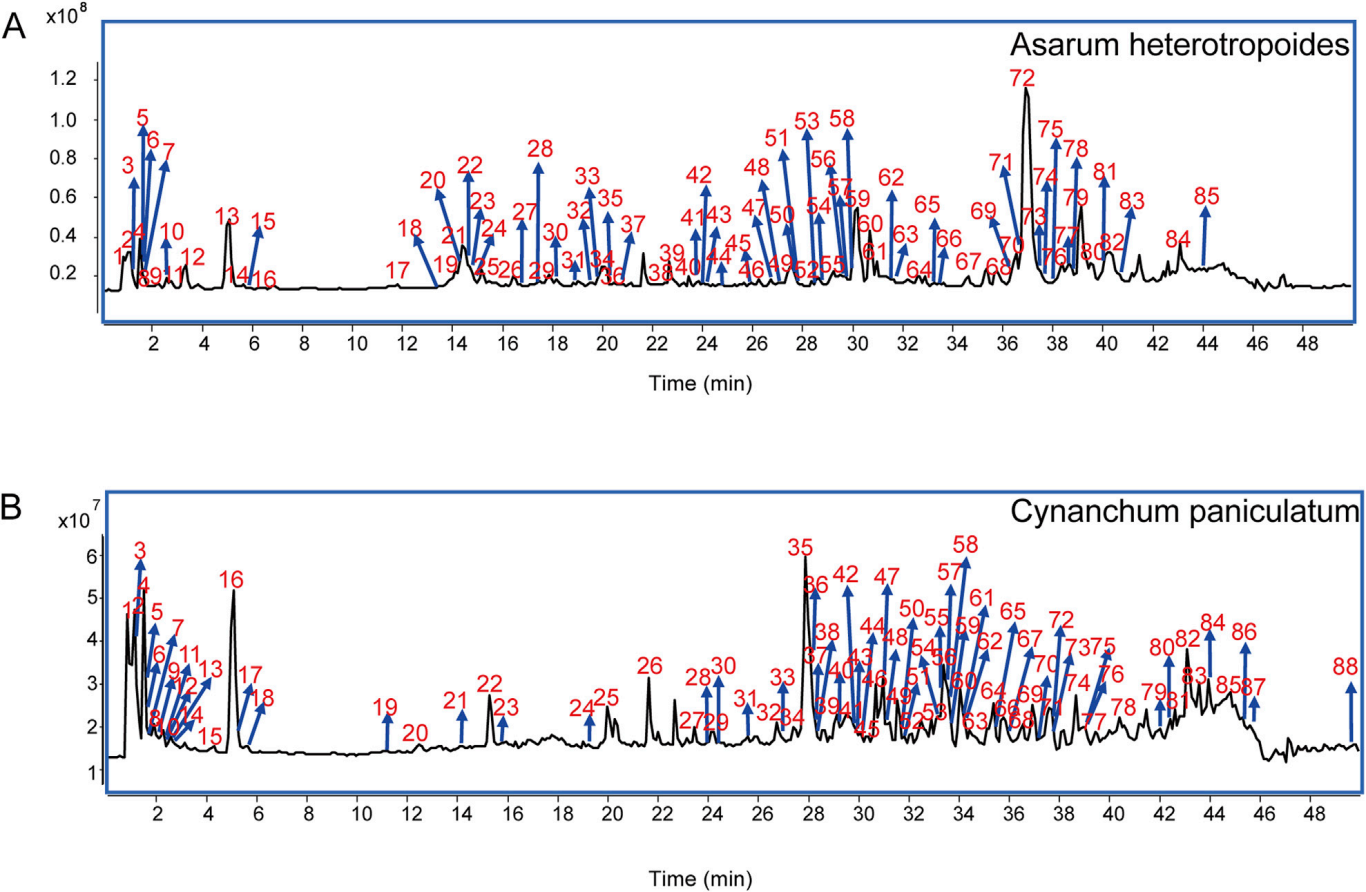
Total ion chromatograms of AH **(A)** and CP **(B)** in positive ion mode.

The chemical compositions of AH and CP were analyzed using LC-HR-Q-TOF-MS/MS. As a result, 91 compounds were identified in AH, comprising 32 nitrogen-containing compounds, 28 volatile oils, 11 organic acids, 6 coumarins, 5 flavonoids, 3 lignans, and 6 other compounds. Notably, ortho-hydroxyacetophenone, 4-hydroxyacetophenone, vanillic acid, and asarinin were confirmed by comparison with their respective chemical standards. The total ion chromatogram (TIC) of AH in positive ion mode is presented in Figure 5A, with detailed compound information listed in Table 2. For negative ion mode, the TIC and compound information are shown in Supplementary Figure S2 and Supplementary Table S1, respectively. Similarly, 90 compounds were identified from CP, including 22 steroidal compounds, 24 nitrogen-containing compounds, 14 volatile oils, 10 organic acids, 8 saccharides, 2 lignans, and 10 other compounds. Among these, paeonol was positively identified by comparison with its chemical standard. The TIC of CP extract in positive ion mode is depicted in Figure 5B, and the corresponding compound information is presented in Table 3. For negative ion mode, the TIC and compound information are shown in Supplementary Figure S3 and Supplementary Table S2, respectively. The discussion on the MS/MS fragmentation patterns of compounds in AH and CP is as follows.

**TABLE 2:** Identification of compounds in AH by LC-HR-Q-TOF-MS/MS.

No.	t_R_ (min)	m/z (Error,ppm)	Formula	Fragmentions (m/z)	Identification
1	0.959	175.1194 (-2.57)^H^	C_6_H_14_N_4_O_2_	130.0955,116.0700,112.0863	L-arginine
2	1.072	138.0553 (-2.52)^H^	C_7_H_7_NO_2_	123.0653,122.4088	Anthranilic acid
3	1.123	137.0600 (-2.16)^H^	C_8_H_8_O_2_	120.0783,121.0817	Ortho-hydroxyacetophenone^a^
4	1.508	137.0600 (-2.16)^H^	C_8_H_8_O_2_	120.0297,121.0733	4-hydroxyacetophenone^a^
5	1.553	180.1019 (0.03)^NH4^	C_10_H_10_O_2_	150.0546,124.0504,110.0365	Safrole
6	1.586	166.0863 (-0.27)^H^	C_9_H_11_NO_2_	122.0690,107.0485,151.1898	Phenylalanine
7	1.602	152.0704 (1.36)^H^	C_8_H_9_NO_2_	110.0338,135.0296	Acetaminophen
8	1.681	121.0648 (-0.07)^H^	C_8_H_8_O	107.0724,103.0543	4-methylbenzaldehyde
9	2.182	180.1019 (0.03)^NH4^	C_10_H_10_O_2_	124.0508,110.0624	Isosafrole
10	2.339	229.0319 (-1.34)^H^	C_5_H_4_N_6_O_5_	138.9636,122.0160,111.8917	6,8-dinitro-3,5-dihydrotetrazolo [1,5-a]pyridin-5-ol
11	2.668	166.0863 (-0.27)^H^	C_9_H_11_NO_2_	122.0855,108.0424,107.0496	Dimethylanthranilate
12	3.413	353.0847 (5.7)^H^	C_16_H_16_O_9_	177.0058,160.9132,118.9029	4-methylumbelliferyl glucuronide
13	5.033	200.0478 (-5.05)^H^	C_12_H_7_O_3_	157.0412,129.0443	6-formylnaphthalene-2-carboxylate
14	5.625	205.0969 (1.25)^H^	C_11_H_12_N_2_O_2_	146.8926,132.0806,118.0647	Tryptophan
15	5.691	188.0707 (-0.51)^H^	C_11_H_9_NO_2_	188.0707,118.0647	3-indoleacrylic acid
16	6.856	136.0617 (0.53)^H^	C_5_H_5_N_5_	120.0381,107.0746	Adenine
17	11.657	330.1699 (0.26)^H^	C_19_H_23_NO_4_	207.0798,177.0787,164.8723,150.0901	Reticuline
18	13.885	379.1000 (6.24)^H^	C_18_H_18_O_9_	217.8755,189.8638,161.8689,185.0418,171.9442	Geshoidin
19	14.000	177.0545 (0.68)^H^	C_10_H_8_O_3_	151.0544,111.0370,134.0348	Hymecromone
20	14.113	147.0440 (0.38)^H^	C_9_H_6_O_2_	118.0407,102.0479	2-benzofurancarboxaldehyde
21	14.403	273.0757 (0.18)^H^	C_15_H_12_O_5_	181.0626,155.0234,153.0177,147.0438,137.9719	(2S)-naringenin
22	14.447	314.1758 (-2.32)	C_19_H_24_NO_3_^+^	209.0954,167.0830,179.0888,153.0692	Magnocurarine
23	14.683	344.1856 (0.10)^H^	C_20_H_25_NO_4_	192.1013,162.0670,138.0625,108.0558	Cilomilast
24	14.906	314.1758 (-2.32)	C_19_H_24_NO_3_^+^	209.0950,167.0799,179.0884,153.0698	Lotusine
25	15.251	177.0545 (0.68)^H^	C_10_H_8_O_3_	121.0261,109.9687,105.0331	7-methoxycoumarin
26	16.461	236.1643 (0.87)^H^	C_14_H_21_NO_2_	165.0687,121.0644	Spectraban
27	16.694	344.1856 (0.10)^H^	C_20_H_25_NO_4_	207.0785,177.0749,147.8670,139.9561	Laudanine
28	17.584	231.0626 (-0.43)^H^	C_9_H_6_N_6_O_2_	148.9010,110.9477	4-[5-(Pyridin-3-yl)-1,2,4-oxadiazol-3-yl]-1,2,5-oxadiazol-3-amine
29	17.881	353.1207 (6.80)^H^	C_17_H_20_O_8_	207.9798,177.8574,164.8723,146.8601	RhytidchromoneD
30	18.153	314.1758 (-2.32)	C_19_H_24_NO_3_	209.0950,167.0832,179.0903,153.0691	(R)-oblongine
31	19.196	236.1643 (0.87)^H^	C_14_H_21_NO_2_	123.0438,107.0485	Meprylcaine
32	19.398	201.1634 (1.88)^H^	C_15_H_20_	187.1392,159.1155,145.0995,131.0846	3,4-dihydrocadalene
33	19.479	268.1328 (1.51)	C_17_H_18_NO_2_	251.1069,219.0783,236.0822,191.0844	Unknown
34	20.165	435.1289 (-0.75)^H^	C_21_H_22_O_10_	273.0748,181.0637,153.0179	(2S)-naringenin-5-O-beta-D-glucopyranoside
36	20.238	273.0757 (0.18)^H^	C_15_H_12_O_5_	181.0615,155.0236,153.0179,147.0444,137.8857	(2R)-naringenin
35	20.255	597.1824 (-1.68)^H^	C_27_H_32_O_15_	435.1288,273.0756	(2R)-naringenin5,7-di-O-glucoside
37	20.467	278.1746 (1.70)^H^	C_16_H_23_NO_3_	128.8733,112.9887,152.9022	Cordypyridone B
38	22.487	215.0678 (0.34)^Na^	C_11_H_12_O_3_	175.0678,144.0570,114.9631	Myristicin
39	22.836	304.1883 (0.05)^Na^	C_16_H_27_NO_3_	222.0666,179.0847,165.0698,205.0649	Scalusamide A
40	23.237	304.1883 (0.05)^Na^	C_16_H_27_NO_3_	248.8163,164.0674	3,3-dimethyl-1-[(2S)-2-pentanoylpyrrolidin-1-yl]pentane-1,2-dione
41	23.814	282.1488 (0.21)^NH4^	C_18_H_16_O_2_	265.1211,250.0973,235.0750,219.0797	Unknown
42	23.839	265.1223 (0.02)^H^	C_16_H_18_O_2_	153.0688,108.9738,122.9137	1,2-bis(3-methylphenoxy)ethane
43	24.071	304.1883 (0.05)^Na^	C_16_H_27_NO_3_	206.8651,164.0700,136.8765	3-acetyl-5-hydroxy-4,5-dimethyl-1-octyl-2-pyrrolone
44	24.696	304.1883 (0.05)^Na^	C_16_H_27_NO_3_	231.8412,180.8673	Ethyl1-(3-cyclopentylpropanoyl)piperidine-4-carboxylate
45	25.939	336.1229 (0.40)^H^	C_20_H_17_NO_4_	320.0908,292.0961,184.9388	N-(biphenyl-4-ylmethyl)-3-hydroxy-6-methyl-4-oxo-4H-pyran-2-carboxamide
46	26.254	387.1413 (6.55)^H^	C_21_H_22_O_7_	302.8310,276.8630,202.8263,176.0397	Sen-byakangelicol
47	26.749	291.1295 (1.42)^H^	C_10_H_18_N_4_O_6_	247.0652,203.0687,159.0353	L-argininosuccinic acid
48	27.021	387.1413 (6.55)^H^	C_21_H_22_O_7_	289.1100,188.8615,161.0210	Edultin
49	27.426	226.1799 (1.13)^H^	C_13_H_23_NO_2_	144.8945,100.9319	Cyclohexyln-cyclohexylcarbamate
50	27.615	183.1012 (2.04)^H^	C_10_H_14_O_3_	168.0745,153.0537,125.0594,137.0592,152.0812	3,4,5-trimethoxytoluene
51	27.665	205.0832 (0.17)^H^	C_8_H_8_N_6_O	137.9020,122.9628,106.9811	2-[(e)-(2H-tetrazol-5-ylhydrazinylidene)methyl]phenol
52	27.688	168.0778 (1.77)^H^	C_9_H_11_O_3_	152.0611,109.0283,137.0060	(3,4-dimethoxyphenyl)methanolradical
53	28.561	179.0700 (1.52)^H^	C_10_H_10_O_3_	135.9491,108.9598,121.0283	Trans-4-methoxycinnamic acid
54	28.646	308.0557 (-1.14)^H^	C_17_H_9_NO_5_	222.0650,278.0566,250.0593,280.0596,252.0642	17-hydroxy-3,5-dioxa-11-azapentacyclo [10.7.1.02,6.08,20.014,19]icosa-1(19),2(6),7,12(20),13,15,17-heptaene-9,10-dione
55	29.223	183.1013 (1.49)^H^	C_10_H_14_O_3_	168.0745,153.0537,125.0594,137.0592,152.0812	2,4,6-trimethoxytoluene
56	29.616	228.1955 (1.34)^H^	C_13_H_25_NO_2_	158.0950,144.0575,100.9321	Cyclohexyl-carbamic acidhexylester
57	29.634	250.1774 (0.28)^H^	C_11_H_19_N_7_	166.1208,155.8579,112.8977	Metazine
58	30.108	209.0809 (-0.31)^H^	C_11_H_12_O_4_	176.0447,161.0231	2-methoxyl-methylenedioxypropiophenone
59	30.182	308.0557 (-1.14)^H^	C_17_H_9_NO_5_	222.0637,278.0567,250.0595,280.0616,252.0634	7-hydroxy-3,5-dioxa-11-azapentacyclo [10.7.1.02,6.08,20.014,19]icosa-1(20),2(6),7,12,14,16,18-heptaene-9,10-dione
60	30.815	338.0665 (-1.74)^H^	C_18_H_11_NO_6_	294.0460,265.0489,250.0261,206.0595	4-[(z)-[2-(1,3-benzodioxol-5-yl)-5-oxo-1,3-oxazol-4-ylidene]methyl]benzoic acid
61	30.983	318.3005 (-0.72)^H^	C_18_H_39_NO_3_	192.8404,164.8297,136.9307	Phytosphingosine
62	31.506	205.0969 (1.25)^H^	C_11_H_12_N_2_O_2_	176.0463,122.0709	Ethotoin
63	31.512	195.0652 (-0.08)^H^	C_10_H_10_O_4_	167.0330,138.9622,123.0408	Kakuol
64	32.667	294.0760 (0.29)^H^	C_17_H_11_NO_4_	279.0521,251.0571,264.0656,236.0693	Aristolactam I
65	33.292	318.3005 (-0.72)^H^	C_18_H_39_NO_3_	192.8434,164.8287,136.9309	2-aminooctadecane-1,3,4-triol
66	33.462	302.3052 (0.52)^H^	C_18_H_39_NO_2_	246.8158,176.9090,106.0860	Sphinganine
67	34.616	222.1850 (1.09)^H^	C_14_H_23_NO	101.9493,152.1066,191.0324	N-isobutyl-2E,4E,8Z-decatrienamide
68	35.844	219.1741 (1.11)^H^	C_15_H_22_O	178.0759,150.0992,122.0691,163.1103,123.0798	Nootkatone
69	36.445	250.2165 (0.16)^H^	C_16_H_27_NO	140.8710,112.9899,100.0754	(2E,4E)-1-(pyrrolidin-1-yl)dodeca-2,4-dien-1-one
70	36.558	219.1741 (1.11)^H^	C_15_H_22_O	191.0859	Longiverbenone
71	36.564	224.2013 (-1.83)^H^	C_14_H_25_NO	167.0813	Pellitorine
72	37.175	249.2077 (4.09)^H^	C_16_H_26_NO	178.1300,151.1344,155.1157	N-methylmeptazinol
73	37.334	248.2014 (-2.06)^H^	C_16_H_25_NO	167.8590,152.1068	N-isobutyl-2E,4E,8Z,10E-dodecatetraenamide
74	37.576	337.1075 (-1.34)^H^	C_20_H_16_O_5_	321.0957,267.0612,237.0545	Psoralidin
75	37.911	248.2014 (-2.06)^H^	C_16_H_25_NO	167.1264,152.1066	N-isobutyl-2E,4E,8Z,10Z-dodecatetraenamide
76	38.392	250.2165 (0.16)^H^	C_16_H_27_NO	153.1093,127.0939,116.0588	Dodeca-2E,4E,8Z-trienoic acidisobutylamide
77	38.522	248.2014 (-2.06)^H^	C_16_H_25_NO	167.8582,152.1066	N-isobutyl-2E,4Z,8Z,10E-dodecatetraenamide
78	38.714	284.1986 (-1.37)^H^	C_16_H_27_O_4_	171.8515,116.0529,128.8711	Monododecylmaleate
79	39.258	274.2171 (-2.05)^H^	C_18_H_27_NO	120.0886,107.0853	8-acetyl-2-(dipropylamino)tetralin
80	39.620	296.1987 (-1.66)^H^	C_17_H_27_O_4_	196.7978,153.9014,127.0712	(E)-5-cyclohexyl-2-[2-[(2-methylpropan-2-yl)oxy]-2-oxoethyl]pent-2-enoate
81	40.125	252.2326 (-1.63)^H^	C_16_H_29_NO	154.1219,112.0753,128.1425,102.0904	(2E,4E)-N-isobutyl-2,4-dodecadienamide
82	40.198	274.2171 (-2.05)^H^	C_18_H_27_NO	120.0534,107.0491	7-(N,N-Dipropylamino)-5,6,7,8-tetrahydronaphtho (2,3-b)dihydro-2,3-furan
83	40.509	276.2324 (-0.76)^H^	C_18_H_29_NO	176.1107,146.0701,107.0850	(1S,2R)-5-methoxy-1-methyl-N,N-dipropyl-1,2,3,4-tetrahydronaphthalen-2-amine
84	42.875	359.1265 (3.59)^H^	C_23_H_18_O_4_	345.1991,253.1547,177.9723,147.0110	7-(benzyloxy)-3-(4-methoxyphenyl)-4H-chromen-4-one
85	44.208	415.0429 (4.69)^H^	C_23_H_10_O_8_	268.0054,241.9654,165.0678,149.0265	5-[4-[(1,3-dioxo-2-benzofuran-5-yl)oxy]benzoyl]-2-benzofuran-1,3-dione

Na, [M + Na]^+^; H, [M + H]^+^; NH_4_, [M + NH_4_]^+^.

^a^ The compounds were identified by comparing with reference substances.

**TABLE 3:** Identification of compounds in CP by LC-HR-Q-TOF-MS/MS.

No.	tR (min)	m/z (Error,ppm)	Formula	Fragmentions (m/z)	Identification
1	1.007	469.2150 (2.58)^H^	C_23_H_48_O_8_	207.0872,181.1031	2-[(E)-4-(2-hydroxy-2-tricyclo [9.4.0.03,8]pentadeca-1 (15),3,5,7,9,11,13-heptaenyl)but-2-enyl]tricyclo [9.4.0.03,8]pentadeca-1 (15),3,5,7,9,11,13-heptaen-2-ol
2	1.087	398.1664 (-1.79)^H^	C_14_H_21_N_8_O_6_	180.0641,164.0709	Methyl 3-o-(2-acetamido-2-deoxy-b-D-galactopyranosyl)-a-D-galactopyranoside
3	1.158	365.1061 (-1.95)^Na^	C_12_H_22_O_11_	186.9692,203.0519	Melibiose
4	1.399	365.1061 (4.77)^H^	C_20_H_43_NO_4_	179.1147,164.0699,150.0893	2-[(2S,3R,4R,5R,6R)-4,5-diacetyloxy-6-(acetyloxymethyl)-3-hydroxyoxan-2-yl]oxyacetic acid
5	1.535	268.1045 (-1.76)^H^	C_10_H_13_N_5_O_4_	136.0617,121.0752	Adenosine
6	1.551	182.0814 (-1.27)^H^	C_9_H_11_NO_3_	136.0755,119.0734	D-Thr-OH
7	1.648	294.1547 (0.10)^H^	C_12_H_23_NO_7_	234.9316,147.0539,117.9572	1,2-O-dimethyl-4-[2,4-dihydroxy-butyramido]-4,6-dideoxy-alpha-D-mannopyranoside
8	1.681	276.1420 (-0.13)^H^	C_12_H_21_NO_6_	190.0614,148.9078	Triethanolaminetriacetate
9	1.936	420.198 (-0.82)^H^	C_17_H_29_N_3_O_9_	258.1306,198.1222,126.0581	Ethyl(2S,4R,5R)-5-azido-4-(methoxymethoxy)-6-[5-(methoxymethoxy)-2-methyl-1,3-dioxan-4-yl]oxane-2-carboxylate
10	1.945	201.0732 (-0.68)^H^	C_5_H_8_N_6_O_3_	158.0701,128.9388,113.9639	2-[(E)-[amino-(4-amino-1,2,5-oxadiazol-3-yl)methylidene]amino]oxyacetamide
11	2.145	298.1396 (0.50)^H^	C_13_H_15_N_3_O_5_	179.0685,122.0610	Hippuryl-glycyl-glycine
12	2.256	420.1980 (-0.82)^H^	C_17_H_29_N_3_O_9_	288.1544,203.0967,159.0642	2-[2-[bis(carboxymethyl)amino]ethyl-[2-[carboxymethyl-(3-methyl-2-oxobutyl)amino]ethyl]amino]acetic acid
13	2.321	283.1402 (-5.16)^H^	C_11_H_22_O_8_	223.1172,163.0966,103.0537	(2R,5R)-3,4-bis(methoxymethoxy)-5-(methoxymethoxymethyl)oxolan-2-ol
14	2.530	214.1186 (6.38)^H^	C_11_H_17_O_4_	174.8792,116.9289	2-o-allyl-3,4-O-isopropylidenearabinopyranosylradical
15	4.342	253.1294 (-4.48)^H^	C_10_H_20_O_7_	179.9909,149.9065,123.0985	2,3-butanediolglucoside
16	5.240	200.0478 (-5.05)^H^	C_12_H_7_O_3_	156.0382,128.0163	2-naphthalen-1-yl-2-oxoacetate
17	5.658	188.0706 (0.03)^H^	C_11_H_9_NO_2_	171.0617,143.0721,118.0645,104.0489	3-indoleacrylic acid
18	5.723	297.1557 (-4.41)^H^	C_12_H_24_O_8_	203.9758,149.0712	Caryophyllose
19	11.239	273.1915 (2.27)^H^	C_12_H_24_N_4_O_3_	174.8691,131.0996,130.0973	4-amino-1-[(3-amino-propyl)-isopropyl-carbamoyl]-pyrrolidine-3-carboxylic acid
20	12.490	313.1249 (-8.31)^H^	C_22_H_16_O_2_	236.8742,144.8656,128.8721	6-(4-hydroxy-phenyl)-1-phenyl-naphthalen-2-ol
21	14.174	362.2407 (1.20)^H^	C_16_H_27_N_9_O	169.9317,140.9187,211.8761,126.9461	2-[[4-[2-(dimethylamino)ethylamino]-6-ethyl-1,3,5-triazin-2-yl]amino]-N-ethyl-3-methylimidazole-4-carboxamide
22	15.304	483.1475 (-7.61)^H^	C_29_H_22_O_7_	229.0671,257.0607,215.0571,171.0257	2-oxopropane-1,3-diylbis (3-phenoxybenzoate)
23	15.810	437.2351 (0.78)^H^	C_16_H_32_N_6_O_8_	219.8877,191.0225,147.9832	2-[[1-[2-[1,1-bis(carboxymethylamino)ethyl-methylamino]ethyl-methylamino]-1-(carboxymethylamino)ethyl]amino]acetic acid
24	19.387	399.1408 (0.86)^H^	C_18_H_18_N_6_O_5_	311.0778,178.0652,148.8566,134.9326	N6-methoxy-2-[(2-pyridinyl)ethynyl]adenosine
25	19.965	399.1408 (0.86)^H^	C_18_H_18_N_6_O_5_	353.1377,220.8733,206.1003	N-[3-[4-(hydroxycarbamoyl)phenoxy]propyl]-6-oxo-2-pyrazol-1-yl-1h-pyrimidine-5-carboxamide
26	21.655	701.4939 (6.85)^H^	C_42_H_68_O_8_	557.0080,412.9373	5-[[(1S,3aS,5aR,5bR,7aR,9S,11aR,11bR,13aR,13bR)-9-(5-hydroxy-3-methyl-5-oxo-pentanoyl)oxy-1-isopropyl-5a,5b,8,8,11a-pentamethyl-1,2,3,4,5,6,7,7a,9,10,11,11b,12,13,13a,13b-hexadecahydrocyclopenta [a]chrysen-3a-yl]methoxy]-3-methyl-5-oxo-pentanoic acid
27	23.492	475.3258 (1.57)^H^	C_25_H_46_O_8_	279.8175,221.9363	(5-acetyloxy-3,4-diheptoxy-6-methoxyoxan-2-yl)methylacetate
28	23.814	219.1008 (3.93)^H^	C_12_H_27_NO_2_	165.1682,137.0588,120.9529	Chuanxiongol
29	24.180	297.2206 (2.34)^H^	C_21_H_28_O	221.1308,185.1308,169.1003	Phenol,2,4-bis(1,1-dimethylethyl)-6-(phenylmethyl)-2,4-di-tert-butyl-6-benzylphenol
30	24.197	679.3301 (-0.13)^Na^	C_33_H_52_O_13_	679.3290,517.2774,312.0463,297.2187	Cynapanoside G
31	25.532	308.2213 (2.34)^H^	C_18_H_29_NO_3_	251.1494,193.1422,138.0851,123.0668,109.0517	Betaxolol
32	26.747	291.1297 (0.73)^H^	C_10_H_18_N_4_O_6_	247.0651,176.1423,160.0386	L-argininosuccinic acid
33	27.038	443.1671 (1.28)^Na^	C_22_H_28_O_8_	291.4742,260.8591,230.0843,146.1015,154.0119	(−)-lyoniresinol
34	27.469	266.1721 (1.07)^H^	C_17_H_17_N_2_O	180.9167,154.0762,152.8657	(2S,4S)-4-azido-1-((S)-2,6-diaminohexanoyl)pyrrolidine-2-carbonitrile
35	27.806	167.0702 (0.43)^H^	C_9_H_10_O_3_	125.0588,111.0394,137.0425	Paeonol^*^
36	27.973	262.0157 (5.24)^H^	C_6_H_9_NO_9_	218.1872,202.9778,144.9735	Glycolatenitrogen
37	28.094	167.0702 (0.43)^H^	C_9_H_10_O_3_	153.0692,137.0221,121.0642,111.0388	Isopaeonol
38	28.163	167.0702 (0.43)^H^	C_9_H_10_O_3_	153.0674,123.0697,109.0275	Ethylparaben
39	29.077	979.4513 (-0.40)^Na^	C_47_H_72_O_20_	979.4491,817.0969,673.3171	Komaroside O
40	29.226	250.1773 (0.68)^H^	C_12_H_25_O_5_	193.0993,136.0314	Metazine
41	29.538	250.1773 (0.68)^H^	C12H25O_5_	168.8645,141.0679,113.9636	[4,6-bis(ethylamino)-1,3,5-triazin-2-yl]-propan-2-ylcyanamide
42	29.604	228.1953 (2.23)^H^	C_13_H_25_NO_2_	130.8979,116.9627,102.9469	4-nonanoylmorpholine
43	29.827	250.1773 (0.68)^H^	C_12_H_25_O_5_	168.9394,141.8710,113.9628	Ethyl-(4-ethylamino-6-isopropylamino-[1,3,5]triazin-2-yl)-cyanamide
44	30.084	250.1773 (0.68)^H^	C_12_H_25_O_5_	235.8168,151.9062	8-(6-aminohexyl)-amino-adenine
45	30.379	285.2894 (2.25)^H^	C_17_H_36_N_2_O	173.9206,117.0710	Tetrabutylurea
46	30.665	274.2742 (-0.53)^H^	C_16_H_35_NO_2_	230.2460,106.0859	N-lauryldiethanolamine
47	30.921	979.4513 (-0.40)^Na^	C_47_H_72_O_20_	979.4499,817.3955,673.3178,299.0703	Komaroside U
48	30.990	318.3003 (-0.09)^H^	C_18_H_39_NO_3_	164.8291,150.1119,106.0649	2,2'-((2-(dodecyloxy)ethyl)imino)bisethanol
49	31.416	993.4648 (1.82)^Na^	C_48_H_74_O_20_	933.4630,833.4395	Marstenacisside A3
50	31.607	817.4020 (-4.92)^Na^	C_41_H_62_O_15_	673.3181,383.1164	Glaucoside D
51	31.672	979.4513 (-0.40)^Na^	C_47_H_72_O_20_	979.4501,817.3950,673.3165,299.0700	Achyranthoside C
52	32.170	316.2842 (1.33)^H^	C_18_H_37_NO_3_	246.8668,176.0696,162.8314	N,N-bis(2-hydroxypropyl)dodecanamide
53	32.883	304.2632 (0.96)^H^	C_20_H_33_NO	191.1254,149.0471,248.2005	Fenpropimorph
54	33.147	831.4144 (-0.81)^Na^	C_42_H_64_O_15_	655.3060,297.1291	(+)-divaroside
55	33.341	817.4020 (-4.92)^Na^	C_41_H_62_O_15_	673.3206,543.2544	Cynapanoside C
56	33.376	963.4573 (-1.38)^Na^	C_41_H_62_O_15_	801.4019,657.3241,299.0692	Cynatratoside D
57	33.448	302.3051 (0.85)^H^	C_18_H_39_NO_2_	302.3051,260.2358,246.1843,232.1683,218.1529,190.1215	Sphinganine
58	33.580	963.4573 (-1.38)^Na^	C_47_H_72_O_19_	657.3232,299.0692	Cynatratoside E
59	33.653	817.4020 (-4.92)^Na^	C_41_H_62_O_15_	673.3181,543.2543,383.1164	Cynapanoside F
60	34.094	817.4020 (-4.92)^Na^	C_41_H_62_O_15_	673.3181,543.2543,383.1164	Glaucoside C
61	34.109	977.4718 (-0.16)^Na^	C_48_H_74_O_19_	917.4468,817.4185	Marstenacisside A2
62	34.151	335.219 (0.00)^H^	C_16_H_26_N_6_O_2_	265.0644,249.1262,233.0831,177.8611	2-(6-(isobutylamino)-2-(pentylamino)-9H-purin-9-yl)acetic acid
63	34.454	817.4020 (-4.92)^Na^	C_41_H_62_O_15_	673.3181,543.2543,383.1164	Hirundigoside C
64	35.168	831.4144 (-0.81)^Na^	C_42_H_64_O_15_	671.3278	Cynapanoside E
65	35.321	437.1936 (2.10)^H^	C_23_H_20_N_10_	356.2191,210.9564	3-(1-Methylpyrazol-4-yl)-6-[1-[5-(1-methylpyrazol-4-yl)triazolo [4,5-b]pyrazin-3-yl]ethyl]quinoline
66	35.481	831.4144 (-0.81)^Na^	C_42_H_64_O_15_	655.3441,435.2209	Deoxoglycyrrhizin
67	35.843	303.0629 (7.57)^H^	C_19_H_10_O_4_	199.0295,158.8591,130.9570	3-benzoylnaphtho [1,2-b]furan-4,5-dione
68	35.939	831.4144 (-0.81)^Na^	C_42_H_64_O_15_	441.2081,329.1572	Gitaloxin
69	36.932	801.4041 (-1.18)^Na^	C_41_H_62_O_14_	657.3234,527.2597,383.1818	Cynanoside K
70	37.12	277.1436 (3.35)^H^	C_16_H_22_O_4_	263.7807,235.9707,149.0153,121.0289,105.0333	1,2-benzenedicarboxylic acid
71	37.591	366.3366 (0.15)^H^	C_23_H_43_NO_2_	212.0646,212.0646,117.0681	Semiplenamide A
72	37.690	801.4041 (-1.18)^Na^	C_41_H_62_O_14_	657.3234,527.2597,383.1818	Cynanoside J
73	37.768	279.2317 (0.56)^H^	C_18_H_30_O_2_	199.8904,159.9928,131.0850	Linolenic acid
74	38.656	815.4195 (-0.85)^Na^	C_42_H_64_O_14_	755.3959,715.3654,655.3442	3-O-S2-11α-O-acetyl-l2β-O-tigloyl-tenacigenin B
75	38.730	277.2159 (1.11)^H^	C_18_H_28_O_2_	237.9916,183.0341,143.0845	Stearidonic acid
76	39.010	295.2265 (0.92)^H^	C_18_H_30_O_3_	167.8593,141.9124	13-keto-9Z,11E-octadecadienoic acid
77	39.483	295.2265 (0.92)^H^	C_18_H_30_O_3_	295.2265,238.8623,208.9628,151.0278	2-{2-[4-(1,1,3,3-tetramethylbutyl)phenoxy]ethoxy}ethanol
78	40.394	301.141 (-0.83)^H^	C_14_H_16_N_6_O_2_	244.9672,164.9589,148.9675	8-amino-2-furan-2-yl-[1,2,4]triazolo [1,5-a]pyrazine-6-carboxylic acidbutylamide
79	41.801	291.1297 (0.73)^H^	C_10_H_18_N_4_O_6_	247.1354,231.1099,160.1096,189.0018	(2S)-2-[[amino-[[(4S)-4-amino-4-carboxybutyl]amino]methylidene]amino]butanedioic acid
80	42.503	425.2152 (4.23)^H^	C_22_H_32_O_8_	266.0295,211.0616,152.1410	Didrovaltrate
81	42.598	282.2794 (-0.92)^H^	C_18_H_35_NO	158.0583,102.0910	Oleamide
82	42.807	359.1259 (5.27)^H^	C_23_H_18_O_4_	333.1705,257.9660,213.9605	2-allyl-4,6-dibenzoylresorcinol
83	43.548	284.295 (-0.74)^H^	C_18_H_37_NO	228.3951,158.9754,116.0496	Octadecanamide
84	44.068	415.0432 (3.97)^H^	C_23_H_10_O_8_	268.0060,177.9745,149.0278	5-[4-[(1,3-Dioxo-2-benzofuran-5-yl)oxy]benzoyl]-2-benzofuran-1,3-dione
85	44.796	423.3241 (3.92)^H^	C_29_H_42_O_2_	337.1505,255.1688,215.0875,201.1629	(3R,4S,4aR,6aR,6bS,14aR,14bR)-4-(hydroxymethyl)-4,6a,6b,11,12,14b-hexamethyl-1,2,3,4a,5,6,7,8,14,14a-decahydropicen-3-ol
86	45.406	291.1297 (0.73)^H^	C_10_H_18_N_4_O_6_	247.0667,189.1625,160.0363	Argininosuccinate
87	45.606	471.106 (3.07)^H^	C_27_H_18_O_8_	310.1218,177.1196,162.0399	Methyl 4-[bis(4-hydroxy-2-oxochromen-3-yl)methyl]benzoate
88	49.667	291.1297 (0.73)^H^	C_10_H_18_N_4_O_6_	247.0628,231.1112,160.0414	(N (omega)-L-arginino)succinic acid

Na, [M + Na]^+^; H, [M + H]^+^.

*The compounds were identified by comparing with reference substance.

#### 3.4.2 Fragmentation patterns of main compositions in AH

**FIGURE 6:**
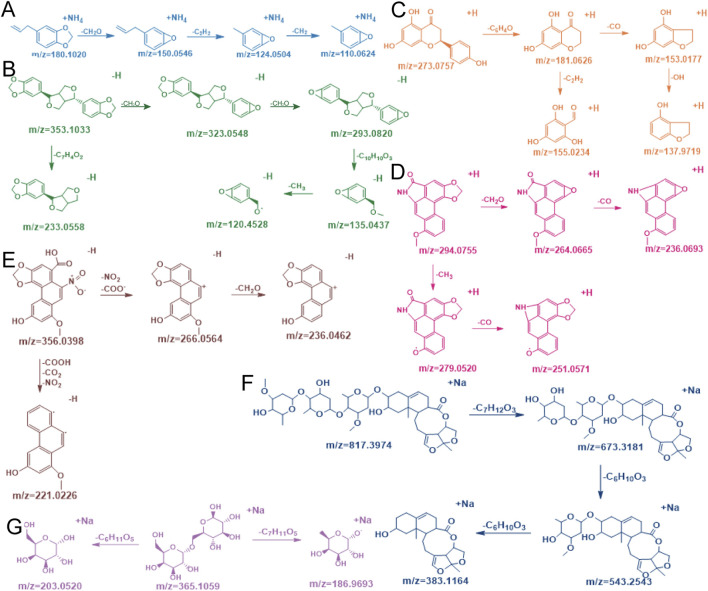
Compound cracking pathways for safrole **(A)** asarinin **(B)** (2S)-naringenin **(C)** aristolactam I **(D)** aristolochic acid **(E)** glaucoside C **(F)** and melibiose **(G)**.

Safrole (Figure 6A) was taken as an example of volatile oils for illustration. The quasi-molecular ion at *m/z* 180 initially underwent methoxy group cleavage to eliminate a molecule of CH_2_O, generating an ion at *m/z* 150. This process might have involved the formation of an allylic carbocation intermediate. Subsequently, the ion at *m/z* 150 underwent rearrangement within the conjugated double bond system, eliminating C_2_H_2_ to yield an ion at *m/z* 124. Finally, this fragment underwent either cleavage of the aromatic ring side-chain CH_2_ group or exocyclic rearrangement to form the stable terminal product ion at *m/z* 110. This fragmentation pathway revealed the stepwise dissociation characteristics of the methoxy group, conjugated double bonds, and aromatic ring structure in the safrole molecule.

Asarinin (Figure 6B) was taken as an example of lignans for illustration. Initially, it started from its quasi-molecular ion at *m/z* 353 and lost a molecule of CH_2_O, generating an ion at *m/z* 323. It then lost another molecule of CH_2_O, forming an ion at *m/z* 293. Subsequently, the ion at *m/z* 293 lost a molecule of C_10_H_6_O_2_, producing an ion at *m/z* 135. Ultimately, the ion at *m/z* 135 underwent another fragmentation, resulting in the loss of a molecule of CH_3_ and yielding an ion at *m/z* 120. In addition, there was another fragmentation pathway that started from the quasi-molecular ion at *m/z* 353, where it lost a molecule of C_7_H_4_O_2_, generating an ion at *m/z* 233 (Hu et al., 2025).

(2S)-naringenin (Figure 6C) was taken as an example of flavonoids for illustration. It started from its quasi-molecular ion at *m/z* 273. By losing a molecule of C_6_H_4_O, this ion transformed into a fragment ion at *m/z* 181. Subsequently, this fragment ion further fragmented and lost a molecule of CO, generating an ion at *m/z* 153. Immediately thereafter, the ion at *m/z* 153 lost an OH group, forming an ion at *m/z* 137. Additionally, there was another fragmentation pathway that started from the fragment ion at *m/z* 181, where it directly lost a molecule of C_2_H_2_, producing an ion at *m/z* 155 (Wen et al., 2014).

Aristolactam I (Figure 6D) was taken as an example of amides for illustration. It started from its quasi-molecular ion at *m/z* 294. By losing a molecule of CH_3_, it generated an ion at *m/z* 279. Subsequently, it lost a molecule of CO, resulting in an ion at *m/z* 251. In addition, there was another fragmentation pathway that started from the quasi-molecular ion at *m/z* 294. In this pathway, the ion lost a molecule of CH_2_O, producing an ion at *m/z* 264. Finally, this ion at *m/z* 264 lost a molecule of CO, yielding an ion at *m/z* 236 (Mao et al., 2017).

Aristolochic acid (Figure 6E) was taken as an example of phenanthrenes for illustration. It began with the quasi-molecular ion at *m/z* 356. By losing a molecule of NO_2_ and a molecule of COO^−^, it generated an ion at *m/z* 266. Subsequently, this ion at *m/z* 266 further fragmented and lost a molecule of CH_2_O, forming an ion at *m/z* 236. Additionally, there was another fragmentation pathway that began with the quasi-molecular ion at *m/z* 356. In this pathway, the ion lost a molecule of COOH, a molecule of CO_2_, and a molecule of NO_2_, producing an ion at *m/z* 221 (Yu et al., 2016).

#### 3.4.3 Fragmentation patterns of major types of compounds in CP

Glaucoside C (Figure 6F) was taken as an example of steroids for illustration. The initial quasi-molecular ion at *m/z* 817 underwent cleavage by losing a molecule of C_7_H_12_O_3_, generating an ion at *m/z* 673. This likely corresponded to the rupture of a glycosidic bond or an ester bond in the molecule, resulting in the detachment of a saccharide or ester group containing 7 carbon atoms, 12 hydrogen atoms, and 3 oxygen atoms. Subsequently, the ion at *m/z* 673 underwent further fragmentation by eliminating a molecule of C_6_H_10_O_3_, producing an ion at *m/z* 543. This step might similarly have involved the cleavage of another saccharide unit or related functional group. Following this, the ion at *m/z* 543 underwent additional fragmentation through the loss of another C_6_H_10_O_3_ molecule, yielding a terminal ion at *m/z* 383. The fragments lost at each step were structural glycosyl units, and these fragmentation processes gradually revealed the structural information of the molecule.

Melibiose (Figure 6G) was taken as an example of saccharides for illustration. The quasi-molecular ion at m/z 365 underwent cleavage at the α-1,6-glycosidic bond, primarily through two distinct fragmentation pathways. In the first pathway, glycosidic bond cleavage was accompanied by elimination of a hexose unit (C_6_H_11_O_6_), resulting in a dehydrated monosaccharide fragment at m/z 185. In the second pathway, direct elimination of the C_6_H_11_O_6_ moiety occurred without hydroxyl group removal, yielding a hydroxyl-retained monosaccharide fragment at m/z 202. These observations suggested that heterolytic cleavage of hydrogen bonds played a critical role in differentiating the fragmentation pathways. Additionally, the intermediate ion observed at m/z 349 (formed via deoxygenation) indicated the loss of a hydroxyl oxygen atom from the sugar ring, generating an unsaturated structure. This structural rearrangement likely facilitated fragmentation pathway branching through intracyclic double bond reorganization.

#### 3.4.4 Component comparison of AH and CP

By comparing the chemical compositions of AH and CP, we could observe significant differences as well as shared components between them. AH primarily comprised nitrogenous compounds, volatile oils, organic acids, coumarins, flavonoids, and lignans. Notably, AH contained unique coumarins and flavonoids that were rare in CP, which exhibited a broad spectrum of pharmacological activities. For example, 7-methoxycoumarin ameliorated hepatotoxicity in rats induced by carbon tetrachloride and spatial memory impairment in ovariectomized Wistar rats induced by scopolamine (Sancheti et al., 2013; Zingue et al., 2018). Naringenin alleviated non-alcoholic fatty liver disease by suppressing the NLRP3/NF-κB pathway and prevented cardiomyopathy through targeting HIF-1α in mice (Wang et al., 2020; Pan et al., 2024). Furthermore, some components in AH exhibited potent toxicity, including aristolactam I, aristolochic acid D, and safrole. Studies demonstrated that aristolactam I accumulated extensively in renal cells and induced nephrotoxicity (Au et al., 2023), while aristolochic acid D triggered lymphocyte infiltration and renal fibroproliferation (Xian et al., 2021). Additionally, safrole exerted hepatotoxicity through the cytochrome P450 enzyme CYP1A2 (Hu et al., 2019). In contrast, the chemical composition of CP mainly included steroidal compounds, nitrogenous compounds, volatile oils, organic acids, saccharides, and lignans. Among them, CP contained unique steroidal compounds and saccharides that were absent in AH, exemplified by glaucoside C and melibiose. Glaucoside C alleviated atopic dermatitis by inhibiting the mitogen-activated protein kinase (Fleitas et al., 2022), while melibiose ameliorated cerebral ischemia/reperfusion injury through regulating autophagic flux (Wu et al., 2021). Despite the chemical differences between AH and CP, they shared common components, such as L-argininosuccinic acid and sphinganine. The results indicated that LC-HR-Q-TOF-MS/MS could differentiate AH and CP from the perspective of chemical compositions.

### 3.5 Electrochemical fingerprint spectra based on Belousov-Zhabotinsky reaction

Although LC-HR-Q-TOF-MS/MS was utilized for analyzing the components of medicinal plants, it also had limitations. On the one hand, it was impossible to identify all the components in medicinal plants. On the other hand, complex data analysis required a considerable amount of time. Therefore, it was necessary to establish a simpler method from the perspective of holistic chemistry, namely, electrochemical fingerprint spectra based on the Belousov-Zhabotinsky reaction. The principle, influencing factors, and model accuracy of this method were as follows.

#### 3.5.1 Principle of electrochemical reactions

Electrochemical fingerprint spectra, as a part of nonlinear chemistry, was capable of characterizing the overall chemical properties of medicinal plants. It arose from oscillations in autocatalytic reactions, revealing fluctuations in the concentrations of certain substances. The principle of this reaction encompassed the consumption of bromide ions (Br^−^), the oxidation of cerium ions (Ce^3+^), and the regeneration of bromide ions (Br^−^). The cycle of bromide ion consumption and regeneration drove the oscillatory system (Wang et al., 2024). The whole components in medicinal plants influenced these reactions, offering novel representations of their chemical properties. For instance, the distinct redox-active components in AH and CP (e.g., ortho-hydroxyacetophenone and paeonol) could influence the oxidation process of Ce^3+^. To ensure the integrity of the phytochemical components, the plant powder was directly involved in the reaction without prior extraction.

#### 3.5.2 Factors influencing Belousov-Zhabotinsky reaction

**FIGURE 7:**
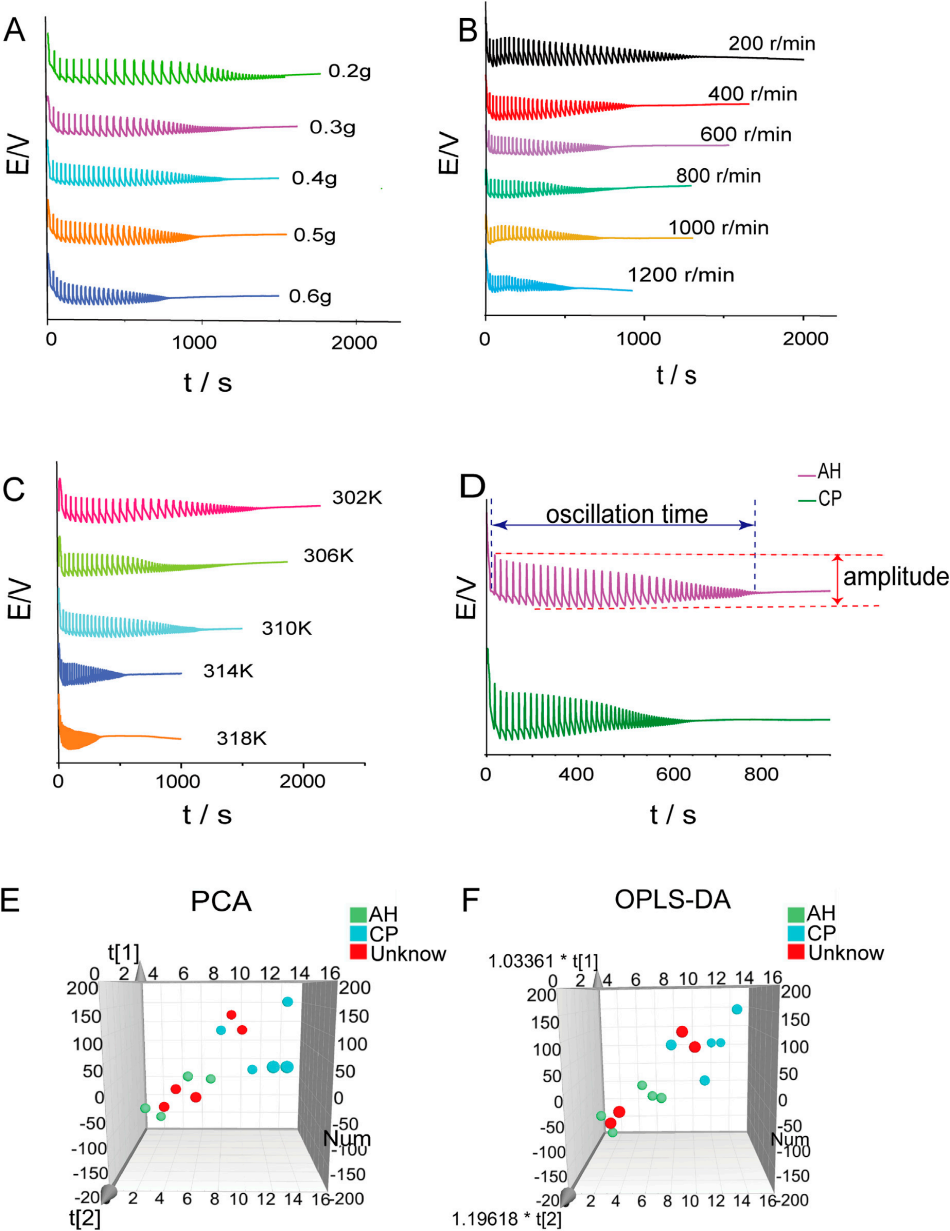
The effects of sample mass **(A)** rotation speed **(B)** and temperature **(C)** on the Belousov-Zhabotinsky oscillation reaction. Electrochemical fingerprint spectra of AH and CP under the same condition **(D)**. Principal component analysis **(E)** and orthogonal partial least squares discriminant analysis **(F)** of AH and CP.

The effects of sample mass, rotation speed, and temperature on the Belousov-Zhabotinsky oscillation reaction were investigated. In Figure 7A, the electrochemical fingerprint spectra of AH powder with varying masses (0.2g, 0.3g, 0.4g, 0.5g, 0.6g) are presented. The characteristic parameters of these spectra were summarized in Supplementary Table S3. Notably, as the mass of the AH powder increased, a discernible trend emerged: the oscillation time gradually decreased, accompanied by a reduction in amplitude. The electrochemical fingerprint spectra of AH powder at stirring speeds ranging from 200 to 1200 r/min (in increments of 200 r/min) are shown in Figure 7B. The characteristic parameters of these spectra were summarized in Supplementary Table S4. As the stirring speed increased, the oscillation time shortened progressively, while the amplitude decreased correspondingly. In Figure 7C, the electrochemical fingerprint spectra of AH at experimental temperatures ranging from 302 to 318 K (in increments of 4 K) were illustrated. The characteristic parameters of these spectra are listed in Supplementary Table S5. As the experimental temperature increased, the oscillation time shortened progressively, while the amplitude decreased correspondingly. It could be seen that the AH powder caused regular changes in the Belousov-Zhabotinsky oscillation reaction. The rotation speed and temperature had a significant influence on this reaction, which should be strictly controlled during the experiment.

#### 3.5.3 Electrochemical fingerprint spectra of AH and CP

The comparison between the electrochemical fingerprint spectra of AH and CP is illustrated in Figure 7D. It could be observed that the oscillation time of AH was significantly longer than that of CP, whereas the maximum amplitude of CP was notably larger than that of AH. To further differentiate the two medicinal plants, the PCA method was employed. The scatter plot is presented in Figure 7E, which shows the separation of AH and CP. The R^2^X and Q^2^ values of this model, at 0.687 and 0.591 respectively, indicated the reliability of the model. Furthermore, the OPLS-DA method was utilized to differentiate between these two medicinal plants (Figure 7F). The result was consistent with that obtained from PCA. The R^2^X, R^2^Y, and Q^2^ values of this model, standing at 0.568, 0.924, and 0.751 respectively, demonstrated the reliability of the outcomes. To assess the accuracy of the model, four unknown samples were analyzed, including two distinct AH samples and two distinct CP samples that had each been independently prepared. The results showed that the unknown samples could be accurately classified into their designated areas, demonstrating a 100% accuracy rate. Compared to LC-HR-Q-TOF-MS/MS, electrochemical fingerprint spectra exhibited the following significant advantages: (1) it allowed for direct analysis of plant powder without extraction, thus simplifying the operation; (2) the analysis time was short, and the data processing was simple. Therefore, it can be concluded that electrochemical fingerprint spectra can be effectively utilized to distinguish between AH and CP.

### 3.6 Integrated analysis of data and methods

AH and CP had very similar appearances, and they were often confused in the market. Given that AH contained toxic ingredients, and both AH and CP had irritating odors and tastes, traditional sensory identification methods, such as nose-sniffing and mouth-tasting, could not accurately distinguish between them. Furthermore, these methods might cause discomfort to the human body. Therefore, we used E-nose and E-tongue to distinguish between the two poisonous and medicinal plants. The E-nose provided the shortest analysis time among all technologies, enabling it to rapidly complete sample testing within 140 s. More importantly, it did not require extraction of samples and the plants could be directly used for analysis, greatly simplifying the operation process. In the PCA and DFA models, the reliability of the E-nose reached 98.912% and 100%, respectively, fully demonstrating its accuracy. The E-nose further disclosed that both AH and CP contained unpleasant ingredients. Specifically, AH included terpinolene, alpha-phellandrene, and camphor, which imparted flavors of anise, plastic, spiciness, and pepper. These ingredients might induce headaches and discomfort. On the other hand, CP contained camphor with a distinct, stimulating peppery taste that could also cause discomfort. At the same time, the E-tongue also revealed that the tastes of components in these two plants were bitter and astringent. In the PCA model, the R^2^ and Q^2^ values of the E-tongue were 0.869 and 0.607, respectively, indicating that the model had good predictive ability and stability. The R^2^X, R^2^Y, and Q^2^ values of the OPLS-DA model were 0.93, 0.936, and 0.895, respectively, further confirming the reliability of the results. Through LC-Q-TOF MS, we found that the bitter and astringent components in AH might be asarinin, N-isobutyl-2E,4E,8Z,10E-dodecatetraenamide, etc., while the bitter and astringent components in CP might be paeonol, etc. Due to the different components of AH and CP, their effects on the Belousov-Zhabotinsky reaction were also different. Based on the electrochemical fingerprint of the reaction, we achieved 100% accurate differentiation between AH and CP. By integrating data from E-nose, E-tongue, LC-HR-Q-TOF-MS/MS, and electrochemical fingerprint spectra, this study provided a diverse perspective based on odor, taste, and chemical composition, thereby providing powerful technical support for accurately distinguishing between AH and CP. It should be noted that the current study focused specifically on AH samples from Anguo City and CP samples from Lu’an City, which represented the mainstream sources of these medicinal plants in the Chinese herbal market. This study was based on a market survey revealing an adulteration practice in which AH (Anguo City) was adulterated with CP (Lu’an City) for illicit profit. Given that the quality of medicinal plants is influenced by geographical origins, growth stages, and plant parts, the impacts of these factors on the current methodology require further systematic and in-depth investigation.

In the field of medicinal plant identification, current techniques such as microscopic identification, DNA barcoding, and near-infrared spectroscopy exhibited distinct characteristics and inherent limitations when applied individually. Microscopic identification enabled rapid and cost-effective differentiation, but some microscopic characteristics lacked sufficient specificity to support accurate identification (Xu et al., 2015). Although DNA barcoding provided specific genetic information, it suffered from low resolution in distinguishing closely related species (Zhu et al., 2022). Near-infrared spectroscopy required minimal sample preparation, but its accuracy was susceptible to interference from factors such as moisture content and particle size (Yin et al., 2019). These limitations highlighted the inadequacy of a single method to address the complex demands of medicinal plant identification. In this study, E-nose, E-tongue, LC-HR-Q-TOF-MS/MS, and electrochemical fingerprint spectra were combined to distinguish the visually similar plants AH and CP. Actually, each method possessed distinct strengths and limitations. E-nose analysis required no sample extraction and could be completed within 3 minutes. However, its detectable targets were restricted to volatile compounds. E-tongue could substitute for human sensory evaluation in detecting the taste of toxic plants, but it was unable to distinguish specific taste components. LC-HR-Q-TOF-MS/MS could resolve chemical components, but data processing required a considerable amount of time. Electrochemical fingerprint spectra offered simple data processing with high accuracy. However, it could only reflect the plant’s electrochemical properties from a holistic perspective. Therefore, through complementary integration of these technologies, the limitations of individual methods were mitigated, and their strengths synergistically enhanced.

## 4 Conclusion

A novel strategy, incorporating dual electronic sensors (DES) and dual fingerprint spectra (DFS), was proposed for the authentication and differentiation of the highly similar poisonous and medicinal plants, AH and CP. The E-nose was utilized to identify 25 odor components in AH and 12 in CP within 140 s, effectively distinguishing the aroma profiles of the two plants. The E-tongue, combined with chemometrics, revealed that bitterness and astringency were the key differentiating tastes. Through the use of LC-HR-Q-TOF-MS/MS for chemical fingerprint spectra, 91 compounds in AH and 90 compounds in CP were identified. To further differentiate AH and CP, electrochemical fingerprint spectra based on the Belousov-Zhabotinsky reaction were established, achieving a 100% accuracy rate. In summary, this study represented the first instance of integrating E-nose, E-tongue, LC-HR-Q-TOF-MS/MS, and Belousov-Zhabotinsky reaction for the authentication and differentiation of highly similar poisonous and medicinal plants.

## Data Availability

The original contributions presented in the study are included in the article/Supplementary Material, further inquiries can be directed to the corresponding authors.
