# Supplementary material for: Discrimination of poisonous and medicinal plants with similar appearance (Asarum heterotropoides vs. Cynanchum paniculatum) via a fusion method of E-nose, E-tongue, LC-HR-Q-TOF-MS/MS, and electrochemical fingerprint spectra
Source: Front Chem. 2025 Apr 29;13:1578126. doi: 10.3389/fchem.2025.1578126 (PMC12069418; doi:10.3389/fchem.2025.1578126)
Supplement: Supplementary file 1 [file DataSheet1.docx]

Supplementary Material

# Supplementary Figures and Tables

## Supplementary Figures

.
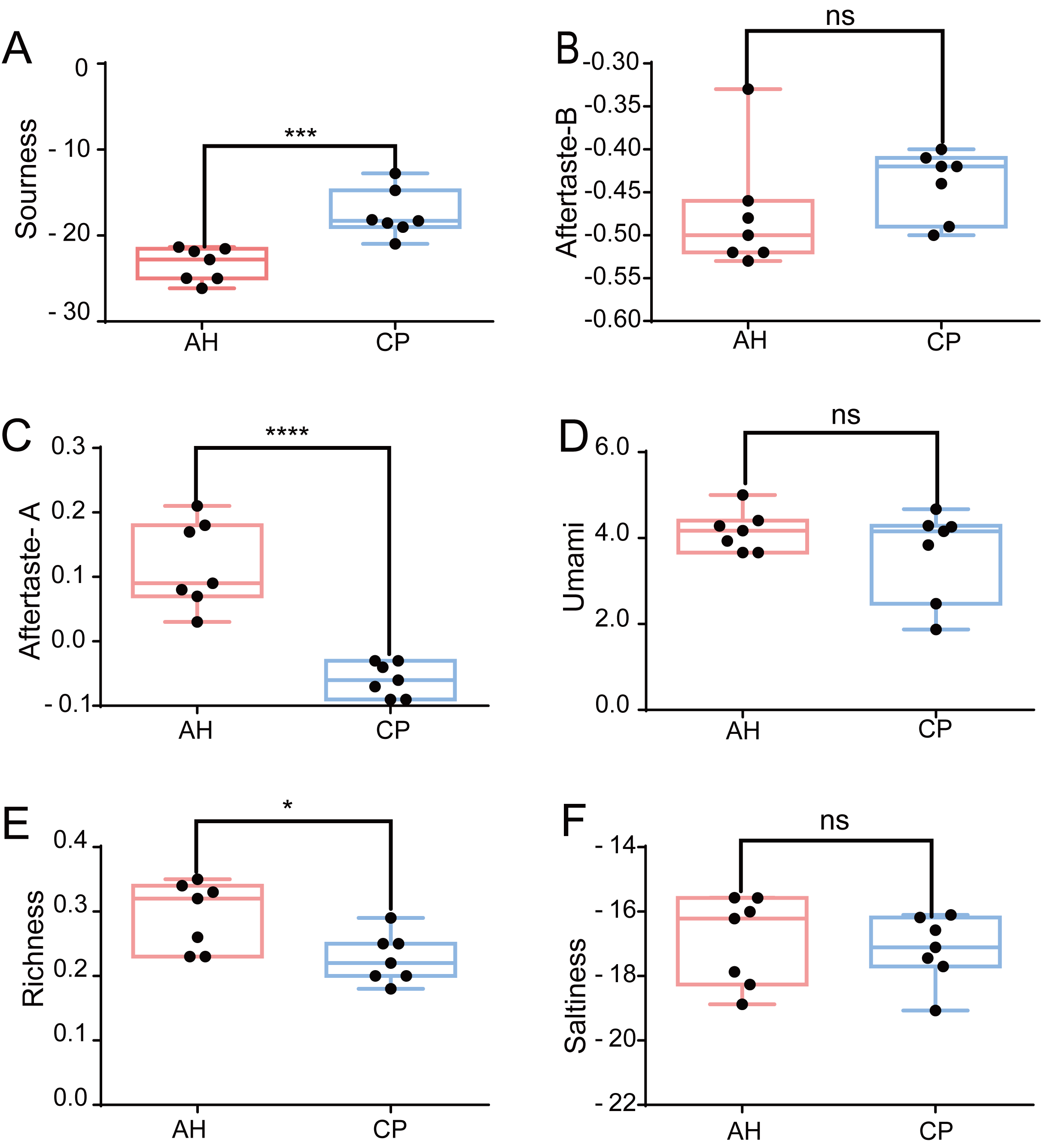


Figure S1 Comparison of sourness (A), aftertaste-B (B), aftertaste-A (C), umami (D), richness (E) and saltiness (F) between AH and CP.

Figure S2 Total ion chromatogram of AH in negative ion mode.

Figure S3 Total ion chromatogram of CP in negative ion mode.

## Supplementary Tables

**Table S1** Compounds of AH identified in negative ion mode by LC-HR-Q-TOF-MS/MS.

| No. | t_R_(min) | m/z(Error,ppm) | Formula | Fragment ions(m/z) | Identification |
| --- | --- | --- | --- | --- | --- |
| 1 | 3.544 | 167.0340(5.85)^a^ | C_8_H_8_O_4_ | 108.0210,152.0068 | Vanillic acid^*^ |
| 2 | 16.968 | 375.1432(4.59)^a^ | C_20_H_24_O_7_ | 251.0546,161.0496,133.9756,233.0555 | Tanegool |
| 3 | 17.562 | 207.0650(6.16)^a^ | C_11_H_12_O_4_ | 192.0386,164.0652,136.9463,177.0216,121.0252 | Methylkakuol |
| 4 | 22.983 | 353.1016(4.13)^a^ | C_20_H_18_O_6_ | 323.0547,293.0730,233.0561,135.0437,120.4528 | Asarinin^*^ |
| 5 | 23.928 | 193.0497(4.80)^a^ | C_10_H_10_O_4_ | 134.0458,149.0597,121.0287,107.0496 | Kakuol |
| 6 | 25.437 | 353.1016(4.13)^a^ | C_20_H_18_O_6_ | 323.0548,293.0820,135.0437,120.4528,233.0558,135.0437,120.4528 | Sesamin |
| 7 | 29.441 | 356.0398(3.89)^a^ | C_17_H_11_NO_8_ | 266.0564,236.0462,221.0226 | Aristolochic acid |

a: [M-H]^－^; * The compounds were identified by comparing with reference substances.

**Table S2** Compounds of CP identified in negative ion mode by LC-HR-Q-TOF-MS/MS.

| No. | t_R_(min) | m/z(Error,ppm) | Formula | Fragment ions(m/z) | Identification |
| --- | --- | --- | --- | --- | --- |
| 1 | 1.083 | 341.1081(2.44)^a^ | C_12_H_22_O_11_ | 270.9346,120.9767,101.0231 | Melibiose |
| 2 | 18.204 | 487.3042(4.74)^a^ | C_29_H_44_O_6_ | 433.3169,374.8110 | Camphoratin A |
| 3 | 29.222 | 221.0805(6.45)^a^ | C_12_H_14_O_4_ | 146.9963,121.0278 | Monobutyl phthalate |
| 4 | 37.170 | 277.1445(0.12)H | C_16_H_22_O_4_ | 147.0079,119.0131,103.0187 | 1,2-benzenedicarboxylic acid |

a: [M-H]^－^.

**Table S3** The impact of sample mass on the electrochemical fingerprint spectrum of AH.

| mass/g | t_induction_/s | t_cessation_/s | t_oscillation_/s | △E_max_/V | E_start_/V | E_cessation_/V |
| --- | --- | --- | --- | --- | --- | --- |
| 0.2 | 12.40 | 1712 | 1699.60 | 0.2321 | 0.1261 | 0.0316 |
| 0.3 | 14.60 | 1222 | 1207.40 | 0.1863 | 0.1125 | 0.0433 |
| 0.4 | 17.70 | 1154 | 1136.30 | 0.1778 | 0.0667 | 0.0569 |
| 0.5 | 18.50 | 996 | 977.80 | 0.1784 | 0.0833 | 0.0329 |
| 0.6 | 20.30 | 819 | 798.90 | 0.1747 | 0.1178 | 0.0512 |

t_induction_-induction time; t_cessation_-cessation time; t_oscillation_-oscillation time; ΔE_max_ -maximum amplitude of oscillation; E_start-potential_-at the start of oscillation; E_cessation-potential_-at the cessation of oscillation.

**Table S4** The impact of rotation speed on the electrochemical fingerprint spectrum of AH.

| Rotation speed  r/min | t_induction_/s | t_cessation_/s | t_oscillation_/s | △E_max_/V | E_start_/V | E_cessation_/V |
| --- | --- | --- | --- | --- | --- | --- |
| 200 | 22.60 | 1370 | 1347.40 | 0.2018 | -0.1070 | -0.0660 |
| 400 | 20.50 | 939 | 918.50 | 0.2047 | -0.0071 | 0.0442 |
| 600 | 14.10 | 835 | 820.90 | 0.1860 | 0.0912 | 0.0612 |
| 800 | 13.80 | 813 | 799.20 | 0.1395 | 0.0930 | 0.0683 |
| 1000 | 9.20 | 751 | 789.90 | 0.0882 | 0.1546 | 0.0678 |
| 1200 | 2.70 | 560 | 577.30 | 0.1299 | 0.2989 | 0.0196 |

t_induction_-induction time; t_cessation_-cessation time; t_oscillation_-oscillation time; ΔE_max_ -maximum amplitude of oscillation; E_start-potential_-at the start of oscillation; E_cessation-potential_-at the cessation of oscillation.

**Table S5** The impact of temperature on the electrochemical fingerprint spectrum of AH.

| temperature/ °C | t_induction_/s | t_cessation_/s | t_oscillation_/s | △E_max_/V | E_start_/V | E_cessation_/V |
| --- | --- | --- | --- | --- | --- | --- |
| 28.8 | 54.90 | 1645 | 1590.10 | 0.2924 | -0.0254 | 0.0388 |
| 32.8 | 47.80 | 1398 | 1350.20 | 0.2587 | -0.0342 | 0.0412 |
| 36.8 | 32.80 | 1231 | 1198.20 | 0.1785 | 0.0539 | 0.0598 |
| 40.8 | 18.60 | 564 | 544.90 | 0.1621 | 0.0366 | 0.0604 |
| 44.8 | 11.50 | 357 | 345.80 | 0.2183 | 0.0967 | 0.0747 |

t_induction_-induction time; t_cessation_-cessation time; t_oscillation_-oscillation time; ΔE_max_ -maximum amplitude of oscillation; E_start-potential_-at the start of oscillation; E_cessation-potential_-at the cessation of oscillation.
